# IPP-DMS: A scalable privacy-preserving data management system for secure and efficient handling of large-scale datasets

**DOI:** 10.1038/s41598-025-32498-6

**Published:** 2025-12-27

**Authors:** Pragya, Sandeep Kumar Mathivanan, Pavan Kumar M. R., Radha Raman Chandan, Kanika Thakur, Usha Moorthy

**Affiliations:** 1 School of Computing Science and Engineering, Galgotias University, Greater Noida, 203201 India; 2 Department of Computer Science and Engineering, Sree Rama Engineering College, Tirupati, India; 3 Department of Computer Science, School of Management Sciences (SMS), Varanasi, 221011 UP India; 4 School of Computer Engineering, Manipal Institute of Technology Bengaluru, Manipal Academy of Higher Education, Manipal, India

**Keywords:** Advanced encryption standard, Artificial intelligence, Data management system, Differential privacy, Role-Based access control, Engineering, Mathematics and computing

## Abstract

The rapid growth of data in various industries has led to a pressing need for innovative solutions that ensure both the security and efficiency of data management systems. As organizations increasingly rely on large-scale datasets for decision-making, ensuring the privacy and integrity of this data becomes a critical challenge. The study introduces the Integrated Privacy-Preserving Data Management System (IPP-DMS), designed to enhance data security, computational efficiency, and user privacy. Unlike conventional systems, IPP-DMS focuses on processing large-scale datasets, such as financial records, customer interactions, and sensor data, employing advanced feature extraction techniques like Gray-Level Co-occurrence Matrix (GLCM) and Principal Component Analysis (PCA) to uncover critical patterns and optimize data handling. The system uses Advanced Encryption Standard (AES) for robust data encryption, differential privacy for data anonymization, and Role-Based Access Control (RBAC) for secure user management. Additionally, an AI-driven anomaly detection module monitors real-time access patterns to ensure compliance with data protection regulations. With an AUC score of 0.91 for anomaly detection, a 0.85-second authentication time, and a 0.45-second access delay, experimental data demonstrate that IPP-DMS works better than conventional systems. In addition, the system provides better scalability and efficiency than PACS, SEISS, and DEIS, processing up to 2000 requests per minute. IPP-DMS provides a scalable, secure, and efficient solution for privacy-preserving data management, setting a new benchmark in the field by integrating advanced methodologies with standard security measures.

**Supplementary Information:**

The online version contains supplementary material available at 10.1038/s41598-025-32498-6.

## Introduction

 Organizations, across various industries, handle sensitive and complex data sets differently today due to the advancements of data management and security systems. The rapid rise in data volumes generated by the sectors of finance, retail, and IoT necessitates innovative approaches toward data management^1^. Organizations not only need to efficiently store and process that amount of data but also protect it from unauthorized access and comply with strict privacy regulations^2^. Integration of Artificial Intelligence (AI) with data management systems becomes a game changer by allowing new accuracy in data analysis, smart pattern recognition, and robust privacy-preserving measures^3^.

Historically, data management systems have been isolated silos where datasets were confined to local systems and could not be easily integrated across different platforms. This often resulted in inefficiencies and missed opportunities for valuable insights^4^. Cloud-based storage systems have partially addressed this by centralizing data management, making it accessible across organizations and enhancing collaborative efforts^5^. The centralization of sensitive data in the cloud has led to new risks such as the possibility of cyberattacks, data breaches, and unauthorized access. The effects of such incidents from financial loss to reputation damage have put forth a significant need for modern data systems to provide comprehensive privacy-preserving mechanisms^6^.

The European General Data Protection Regulation (GDPR) and the California Consumer Privacy Act (CCPA) regulate the processing of sensitive information: strict requirements in handling sensitive data, with full transparency, accountability, and security by organizations. But compliance is strictly adhered to under heavy penalties for non-compliance^7^. Traditional access control mechanisms used to govern data access are mainly Role-Based Access Control (RBAC), which is widely used. But these fall short in dynamic environments, where access should be context-sensitive, such as being allowed to change based on user location, time, or urgency. This has consequently led to the development of more flexible models like Attribute-Based Access Control (ABAC), which considers multiple attributes to define access permissions^8,9^. While ABAC provides increased flexibility, it also introduces complexities that can result in administrative overhead and delays in decision-making, ultimately undermining its effectiveness in fast-paced, dynamic environments^10^.

Encryption remains a cornerstone of data security, protecting data both during transmission and at rest. Traditional encryption methods such as symmetric and asymmetric cryptography provide essential security for stored data but come with limitations. They can be computationally expensive and often unsuitable for real-time applications, especially when dealing with large datasets^11^. Moreover, these encryption methods do not inherently protect against data tampering or ensure the integrity of information^12^. Emerging technologies, such as homomorphic encryption, allow computations to be performed on encrypted data without decryption or exposing secret keys, but the computational burden of these methods presents a significant barrier to widespread adoption in resource-constrained environments^13^.

The increasing integration of AI and Machine Learning (ML) into data management systems further complicates the landscape. AI and ML algorithms allow organizations to extract valuable insights from massive datasets by detecting patterns, predicting trends, and making data-driven decisions^14^. In finance, retail, and IoT, AI models can help in fraud detection, customer behavior analysis, and predictive maintenance^15^. However, the use of AI introduces privacy concerns because these models usually require access to large datasets for training. There is not enough anonymization of data, or there may be breaches during the processing of training data. Such an action could lead to privacy breaches on individuals, especially if the data includes Personally Identifiable Information (PII). Many AI models are “black boxes,” and their decision-making processes are not transparent. Such is a challenge in industries that require accountability and explainability^16^.

Real-time access to data is another critical requirement in modern data management systems. In distributed environments where data is held in multiple places or accessed through different departments, delays in obtaining or sharing information can severely impact decisions. Although centralization of storage through cloud-based systems can mitigate these issues to some extent, latency is very common, especially while dealing with vast amounts of data or in heavily used scenarios^17^. Decentralized approaches, including blockchain, have been proposed for overcoming these kinds of challenges. Blockchain provides distributed ledger systems in a way of secured, tamper-proof storage, and has allowed faster accessibility while maintaining heightened security. Challenges still exist concerning blockchain’s potential adoption in scalable energy consumption-related areas^18^.

Mostly, recent agreements among research scholars and industry experts are looking towards the implementation of integrated multiple technology systems to help resolve these difficulties^19^. Hybrid models combine RBAC and ABAC access control with anomaly detection driven by AI, enabling both data and access control through enhanced security, which can even monitor user behaviors and access patterns constantly to identify emerging security threats against unauthorized access or an insider attack^20^. Similarly, integration of advanced encryption techniques with decentralized frameworks such as blockchain provides a two-layered approach to data security, ensuring privacy and integrity. However, practical deployment of such integrated systems remains complex due to high implementation costs, computational requirements, and the need for specialized expertise^21^.

Another key aspect is the development of privacy-preserving techniques throughout the data processing pipeline. Raw data is often preprocessed and segmented before analysis in AI-driven systems, which may leak private information. Differential privacy is one of the techniques used to add controlled noise to the data, ensuring individual privacy without sacrificing meaningful analysis. However, the challenge is to find a proper balance between privacy and utility since too much noise degrades data quality and the accuracy of the derived insights^22^.

Modern data management systems also take into account the aspects of operational efficiency. Many traditional access control methods require manual intervention and can lead to delay in decision making and add to administrative burdens^23^. Automation of access management processes through AI and ML can help smooth over these procedures, making it faster and more accurate. Access prediction through analytics can be granted based on historical data, user behavior, and contextual factors, which reduces delays and enhances operational efficiency. However, automation also raises ethical and legal concerns, particularly if errors in prediction lead to unauthorized access or the denial of legitimate requests^24^.

The integration of multimodal data sources such as transactional data, sensor data, and customer interactions adds another layer of complexity to data management. While integrating diverse data types can provide richer insights, it also creates challenges related to data format standardization, interoperability, and secure sharing. Initiatives like the Fast Healthcare Interoperability Resources (FHIR) standard have made some progress toward making data interoperability better, though their adoption remains quite limited. Most organizations use proprietary systems that prevent seamless sharing of data.

Therefore, to address the current and future needs of modern organizations, an integrated, privacy-preserving data management system needs to be developed. It would, therefore, integrate AI, cryptography, and decentralized frameworks in such a system so that the sensitive data would be protected, accessible, and usable in real-time. It would also be dynamic and adaptable to different environments with a view to scaling up and down for the diverse requirements of different industries. Through such integration, advanced systems would transcend the constraints of traditional approaches by providing enhanced security for data, improving efficiency in operations, and ensuring regulatory compliance.

The purpose of the study is to develop an Integrated Privacy-Preserving Data Management System (IPP-DMS) that addresses the core challenges of modern data management. The system is developed based on AI-driven anomaly detection, advanced cryptographic techniques, and decentralized technologies like blockchain, in order to create a secure, efficient, and user-friendly data management ecosystem. It enables organizations to fully harness the power of big data while ensuring the protection of sensitive information and compliance with privacy regulations.

With a focus on healthcare imaging data, this study introduces the Integrated Privacy-Preserving Data Management System (IPP-DMS). Robust data management systems that ensure the security, privacy, and integrity of sensitive patient information are needed because of the rapid growth in medical imaging and strict privacy regulations such as HIPAA. In spite of the fact that IPP-DMS is also applicable for financial services and IoT sensor data, our extensive study centers on its use in healthcare imaging environments due to the immense requirement for secure, confidential solutions there.

The enormous growth in data and the increasing problem of unauthorized access introduce a whole range of new challenges to modern data management. Real-time surveillance is facilitated by AI-based anomaly detection mechanisms that detect unusual patterns of access. A hybrid solution for safe, personal data handling is obtained via the combination of blockchain technology to offer decentralized, tamper-proof data storage with AES encryption, which guarantees confidentiality of data.Better security, business efficiency, and compliance with privacy legislation like GDPR and HIPAA are offered via this integration.

The novelty of the proposed system is that it combines blockchain, AI-driven anomaly detection, Role-Based Access Control (RBAC), Advanced Encryption Standard (AES), and differential privacy into a single data management system. Although each of these methods has been applied independently in other systems, their combination in the context of managing enormous amounts of data while maintaining privacy provides a special contribution to the area. When handling complicated datasets, this integrated method guarantees improved security, operational effectiveness, and adherence to privacy laws. The focus of this study is to apply the Integrated Privacy-Preserving Data Management System (IPP-DMS) to healthcare imaging environments, ensuring secure and efficient management of sensitive patient data. Although the system can handle other types of data, this study specifically addresses its application to healthcare imaging, including datasets such as the NIH Chest X-ray and The Cancer Imaging Archive (TCIA).

The main contributions are.

1.  To design an Integrated Privacy-Preserving Data Management System (IPP-DMS) that guarantees the security and confidentiality of sensitive data in cloud-based and distributed environments.
2. To implement advanced cryptographic techniques and decentralized frameworks to protect data from unauthorized access and breaches.
3. To improve operational efficiency by reducing latency and streamlining data sharing and analysis in real-time workflows.
4. To incorporate AI-driven anomaly detection for real-time monitoring of access patterns, robust privacy protection, and regulatory compliance.

Along with a detailed introduction, Section II describes the system design, Section III depicts the experimental results and discussions followed by conclusion in Section IV.

## System design

### Data description

The study utilizes actual healthcare imaging datasets, specifically The Cancer Imaging Archive (TCIA) and the NIH Chest X-ray dataset, which include anonymized patient data commonly used in medical image analysis research. These datasets provide a realistic setting for evaluating the privacy-preserving capabilities of the Integrated Privacy-Preserving Data Management System (IPP-DMS). The system’s effectiveness in securing healthcare data, maintaining privacy, and performing anomaly detection is assessed using medical images such as chest X-rays and CT scans.

Additionally, to demonstrate the versatility of IPP-DMS, the Credit Card Fraud Detection dataset is employed as a secondary use case. This dataset, obtained from (https://www.kaggle.com/datasets/nelgiriyewithana/credit-card-fraud- detection-dataset-2023), contains over 284,807 transactions, with 31 anonymized features (V1-V28, transaction amount, time, etc.). The target variable in this dataset is Class, where 0 indicates legitimate transactions, and 1 indicates fraudulent ones. The dataset is highly imbalanced, with fraudulent transactions making up only 0.172% of the total.The IPP-DMS integrates privacy-preserving mechanisms such as anonymization and encryption to ensure sensitive customer data—like identity and transaction details—remains protected while still allowing accurate fraud detection. This approach ensures compliance with privacy regulations while maintaining effective fraud detection.

**Fig. 1:**
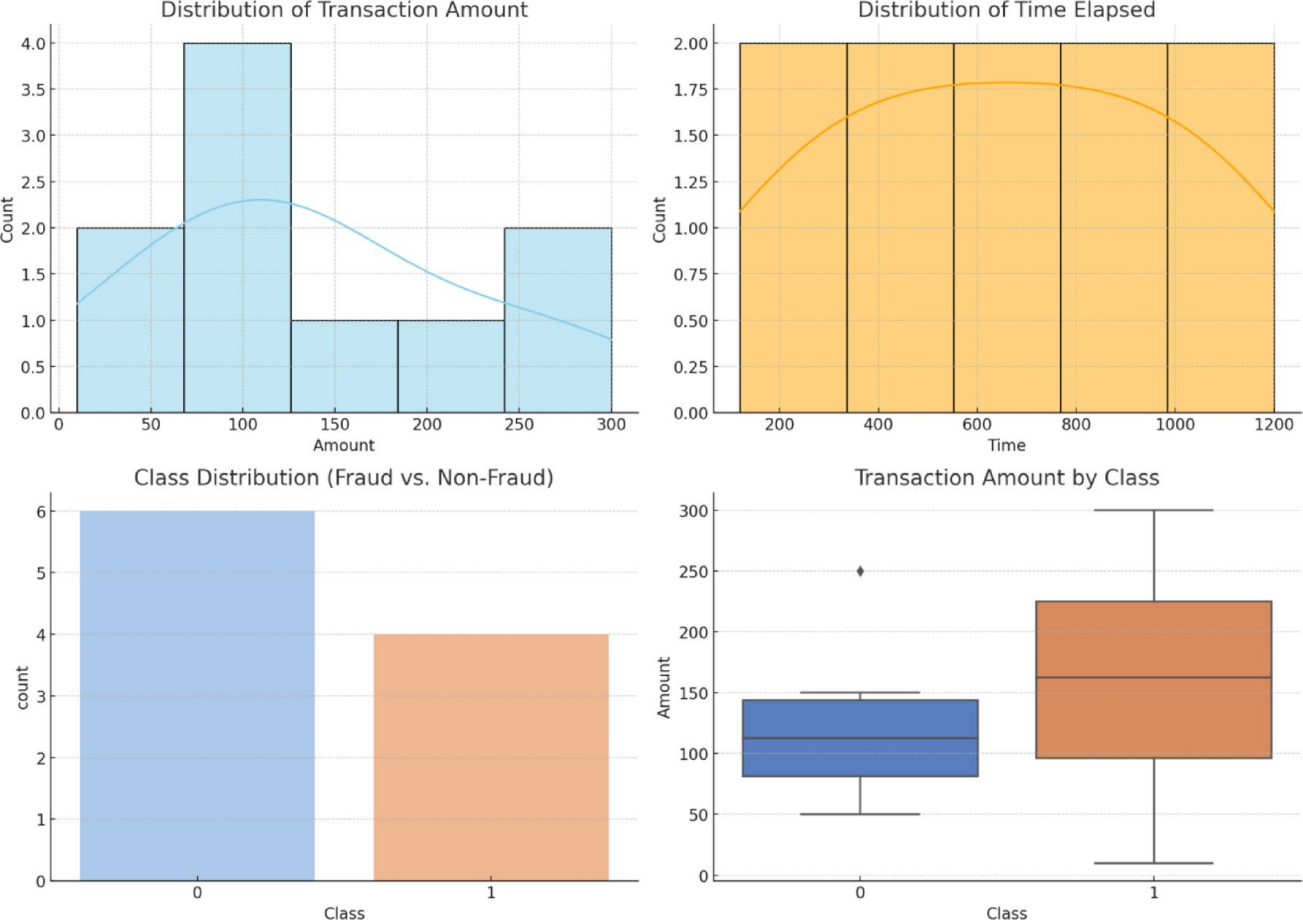
Data Distribution.

Figure 1 includes four plots for the description of characteristics in the transaction data. Distribution of transaction amount, time elapsed, class distributions, transaction amount by class are plotted.

### Data preprocessing

The data preprocessing involves filling the missing values using mean imputation so that there is no gap in the dataset. It addresses the class imbalance because 0.172% of the transactions are fraudulent; SMOTE (Synthetic Minority Over-sampling Technique) is applied to oversample the minority class (fraudulent transactions), balance the dataset, and make it easier to identify fraud cases. The Amount feature, ranging from cents to thousands of dollars, and the anonymized PCA features V1 through V28 are scaled by standardization, making their mean equal to 0 and their standard deviation equal to 1. In this way, all features have an equal influence on the model, which is crucial for logistic regression and neural networks.

**Fig. 2:**
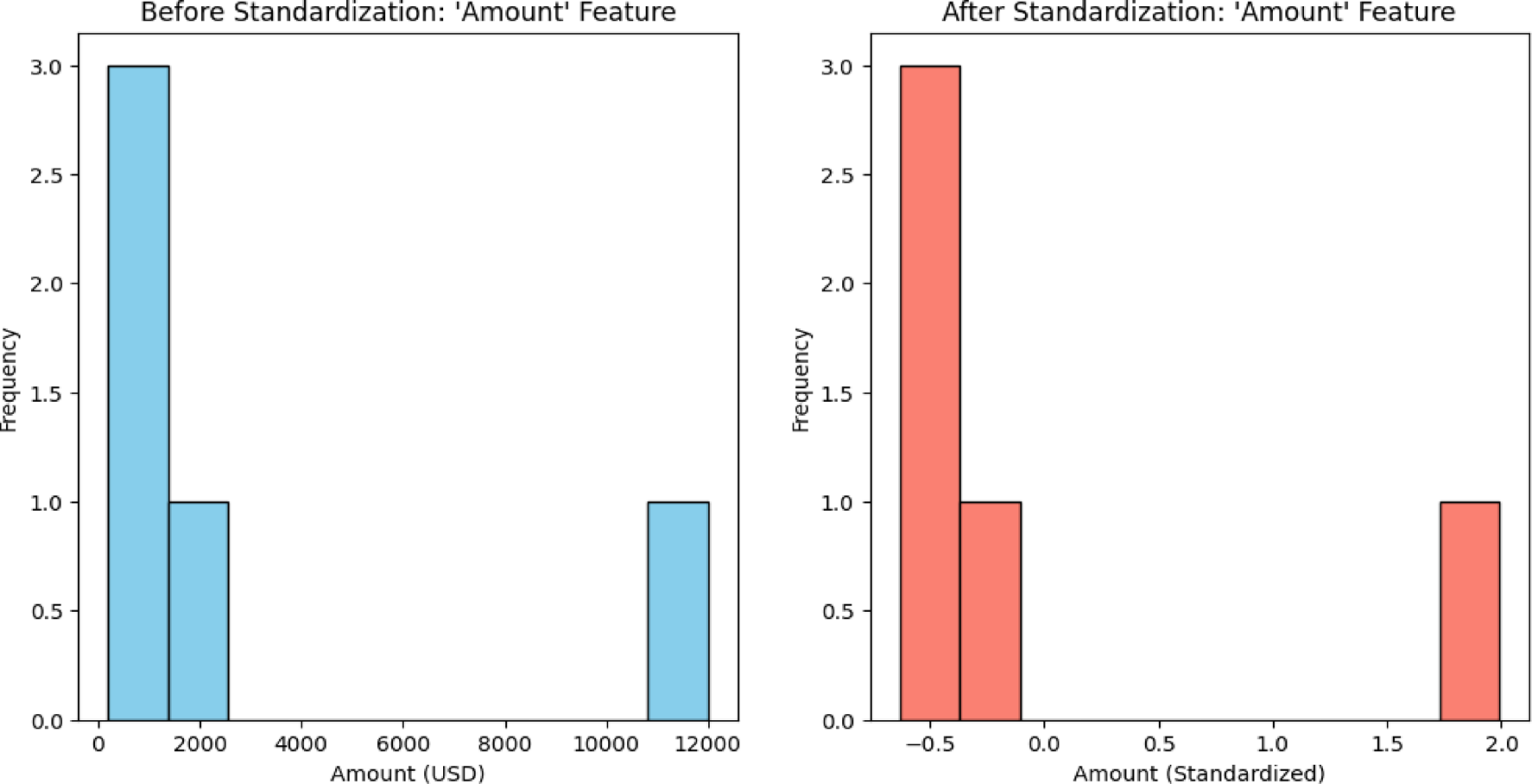
Data Standardization - Amount Feature.

**Fig. 3:**
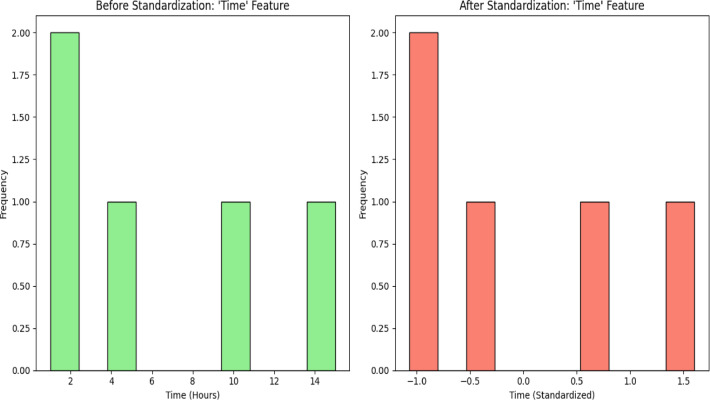
Data Standardization - Time Feature.

We employed the IQR(Interquartile Range) filtering to address outliers. We also examined alternative strategies since we knew of the risk of excluding meaningful, unusual events such as large-scale fraudulent transactions. To help maintain valuable data, capping was utilized, wherein transaction values above the 95th percentile were replaced by the 95th percentile value. To minimize the effects of outliers and retain the integrity of rare fraud transactions, we also considered strong estimators like Median Absolute Deviation (MAD) and robust regression. Outliers on the Time and Amount are detected by means of IQR method. Those that fall out of the 1.5 IQR range are deleted because they tend to distort the predictions of the model. The dataset is then divided into training and testing sets, usually in a ratio of 80 − 20%, to ensure that the model is tested on unseen data to avoid overfitting. Finally, anonymization techniques are applied to the dataset to protect sensitive customer information while enabling effective fraud detection. Before preprocessing, the Amount and Time feature ranges included broad raw values: Amount from small to large, while Time had intervals between single transactions. Standardizing the feature in preprocessing brings Amount and Time to a mean of 0 and a standard deviation of 1. This makes the scale uniform so that no one single feature dominates based on its size for the machine learning models to process. Figures 2 and 3 depicts the standardization of features ‘Amount’ and ‘Time’.

### Proposed security architecture

Four algorithms are used for Integrated Privacy-Preserving Data Management System (IPP-DMS) to ensure the security, confidentiality, and operational efficiency of financial data in cloud-based and distributed environments. These algorithms incorporate advanced cryptographic techniques, decentralized frameworks, anomaly detection, and data sharing integration. In order to address the issues of safe storage, retrieval, and privacy-preserving management of medical images, the IPP-DMS system was created with healthcare imaging as its main use case. Due to the unique security and legal requirements in the healthcare industry, the system’s primary assessment focuses on medical imaging, even if it can be modified to other domains including financial transactions and IoT sensor data. Ethereum was chosen as the blockchain technology for the system due to its security features, tried and tested security features, and ability to employ smart contracts for safe and transparent data storage. Ethereum’s decentralized ledger ensures that transaction information is auditable and irreversible, which makes it the ideal choice for dealing with private data. In addition, Ethereum’s robust support for decentralized applications (dApps) and compatibility with existing technology provided the scale and flexibility the system required. The anomaly detection algorithm was designed to monitor user access patterns to sensitive healthcare imaging data. This AI-driven model identifies unauthorized access attempts or potential data breaches, ensuring compliance with healthcare regulations such as HIPAA.

### Algorithm 1: encryption and decryption

**Fig. 4:**
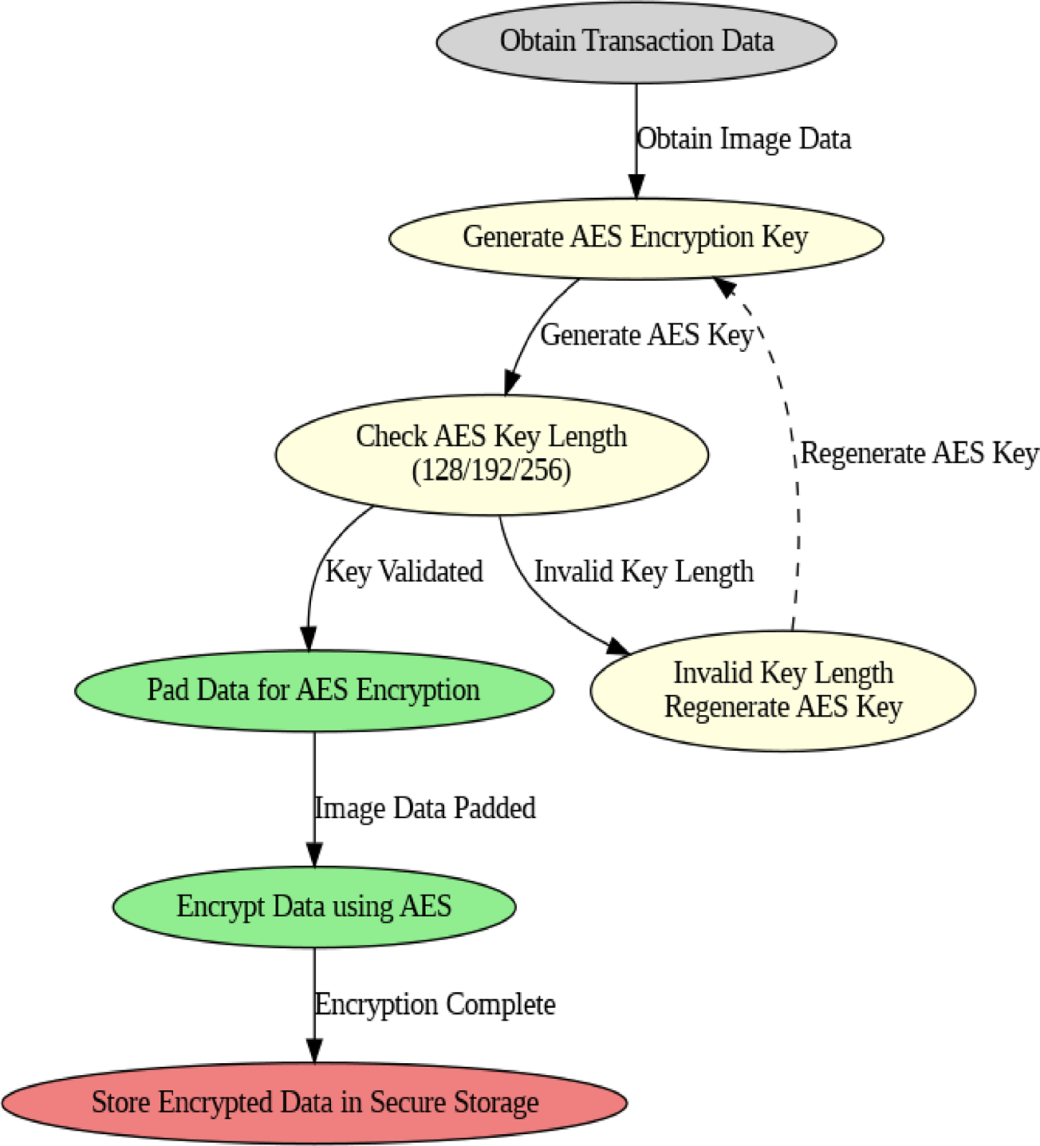
AES Encryption Framework.



The algorithm encrypts data using AES (Advanced Encryption Standard) to protect sensitive financial information. The encryption ensures that data cannot be accessed or read without proper decryption keys (Fig. 4).

### Algorithm 2: Role-Based access control (RBAC) for transaction data

**Fig. 5:**
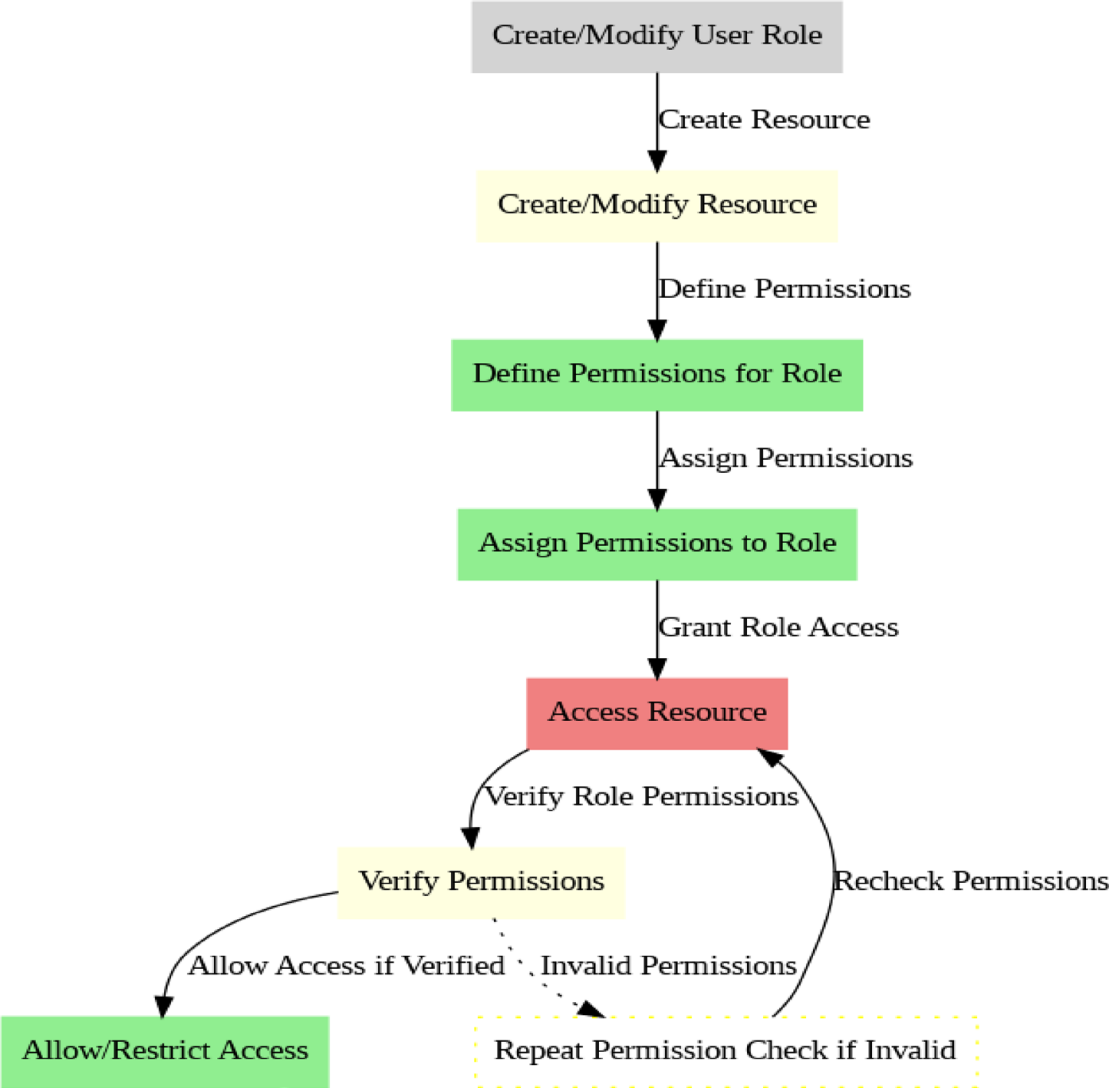
RBAC Process.



Due to its simplicity and efficiency in managing access to confidential information in the system, Role-Based Access Control (RBAC) is used. RBAC is chosen due to its simplicity of use and lower administrative overhead, which is critical in high-performance environments such as healthcare and finance, although more dynamic models such as Attribute-Based Access Control (ABAC) were also considered. ABAC was not utilized in the design since, although it offers more flexibility, it introduces complexities that can affect system scalability and real-time behavior.The algorithm implements Role-Based Access Control (RBAC) to ensure that only authorized users with specific roles can access data. It provides functionalities for creating, modifying, and deleting access control policies for resources (Fig. 5).

### Algorithm 3: anomaly detection for Real-Time monitoring (AI-Based monitoring)

**Fig. 6:**
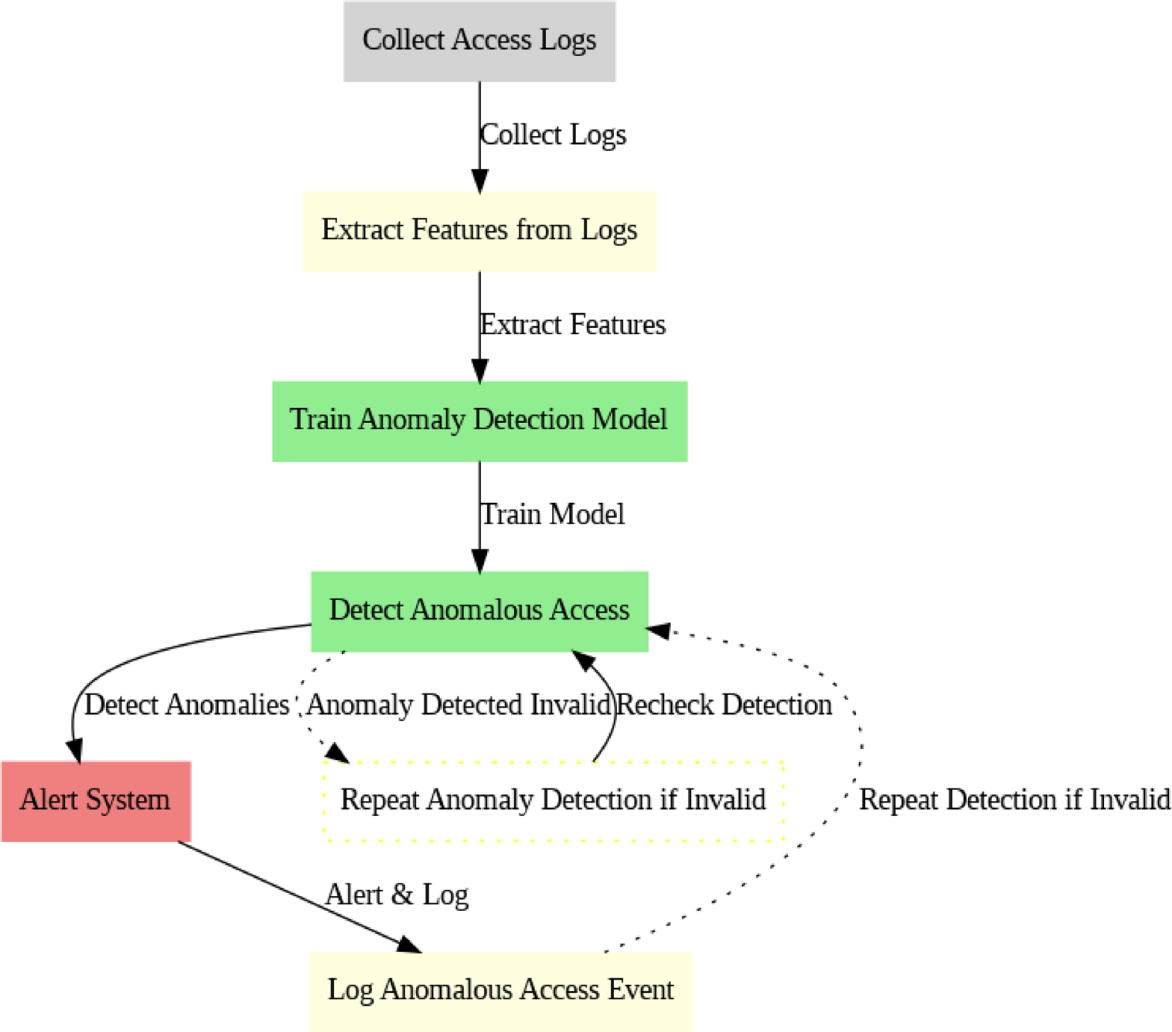
AI Based Monitoring Process.



The algorithm uses an AI-driven model to monitor user access patterns in real-time. The goal is to detect any abnormal access behavior, such as unauthorized access to sensitive transaction data, which may indicate a breach(Fig. 6).

### Algorithm 4 :decentralized data sharing with blockchain

**Fig. 7:**
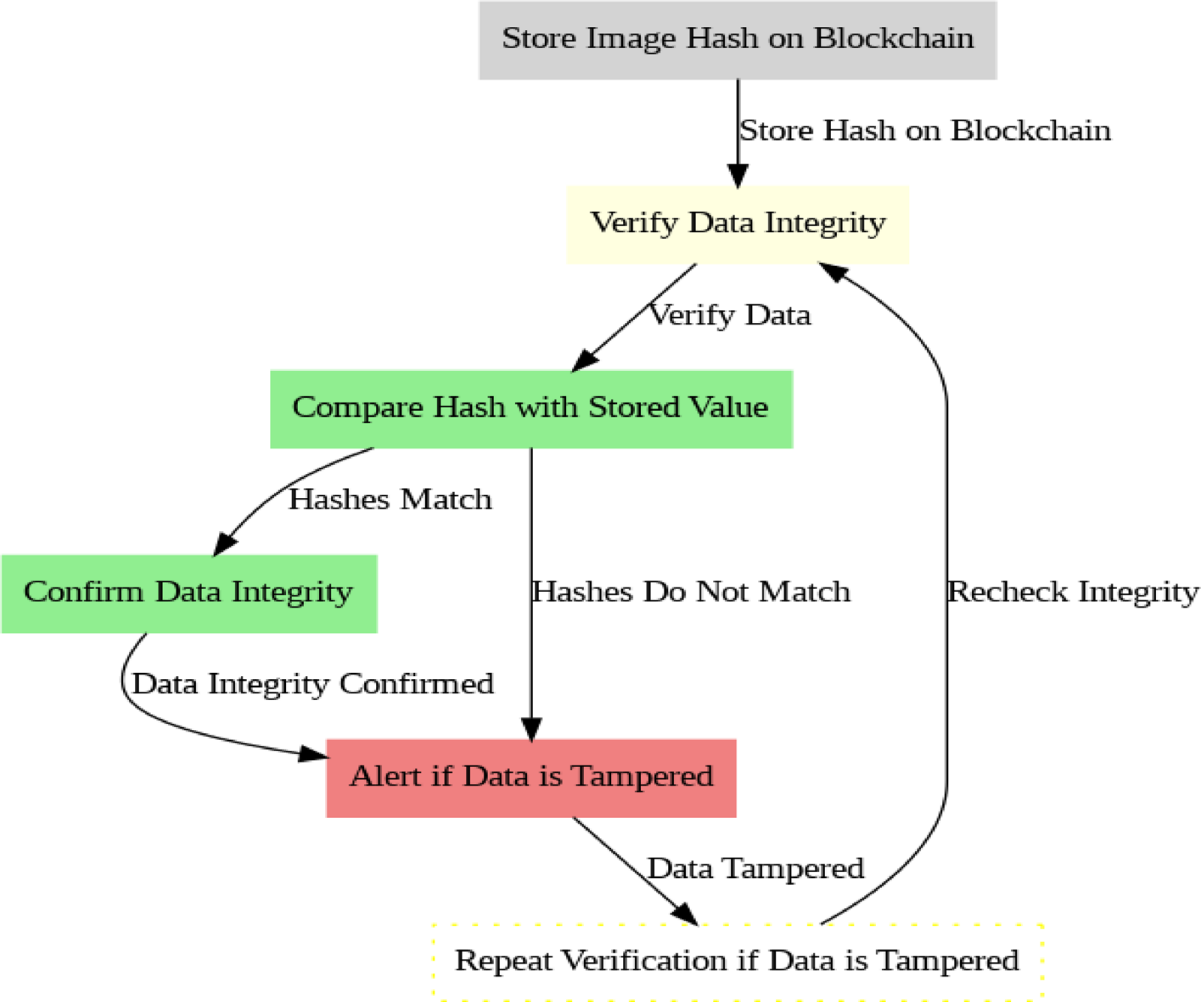
Blockchain Process.



The algorithm incorporates blockchain to enable secure, decentralized data sharing of transaction data across various healthcare providers while ensuring data integrity and privacy(Fig. 7).

**Fig. 8:**
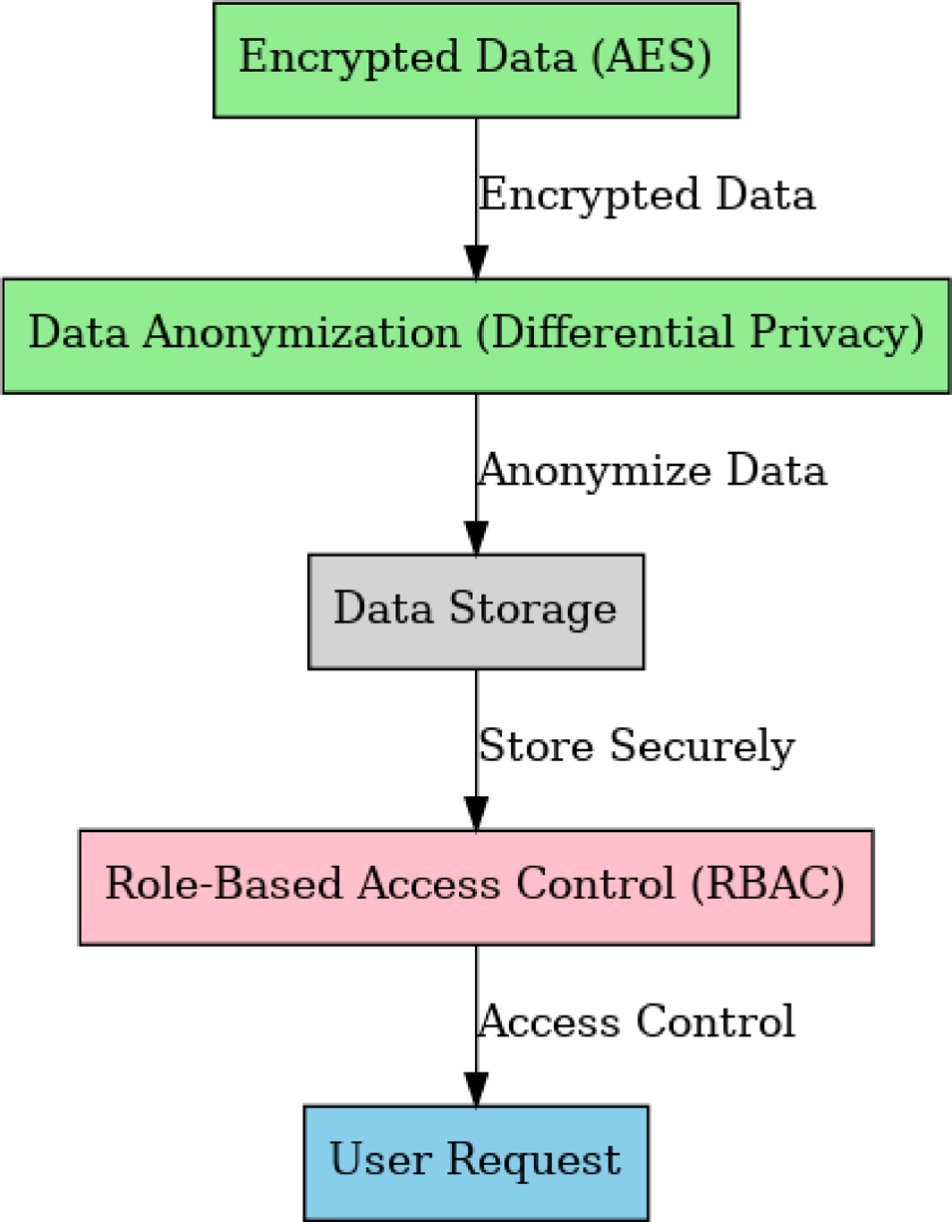
Basic Framework.

**Fig. 9:**
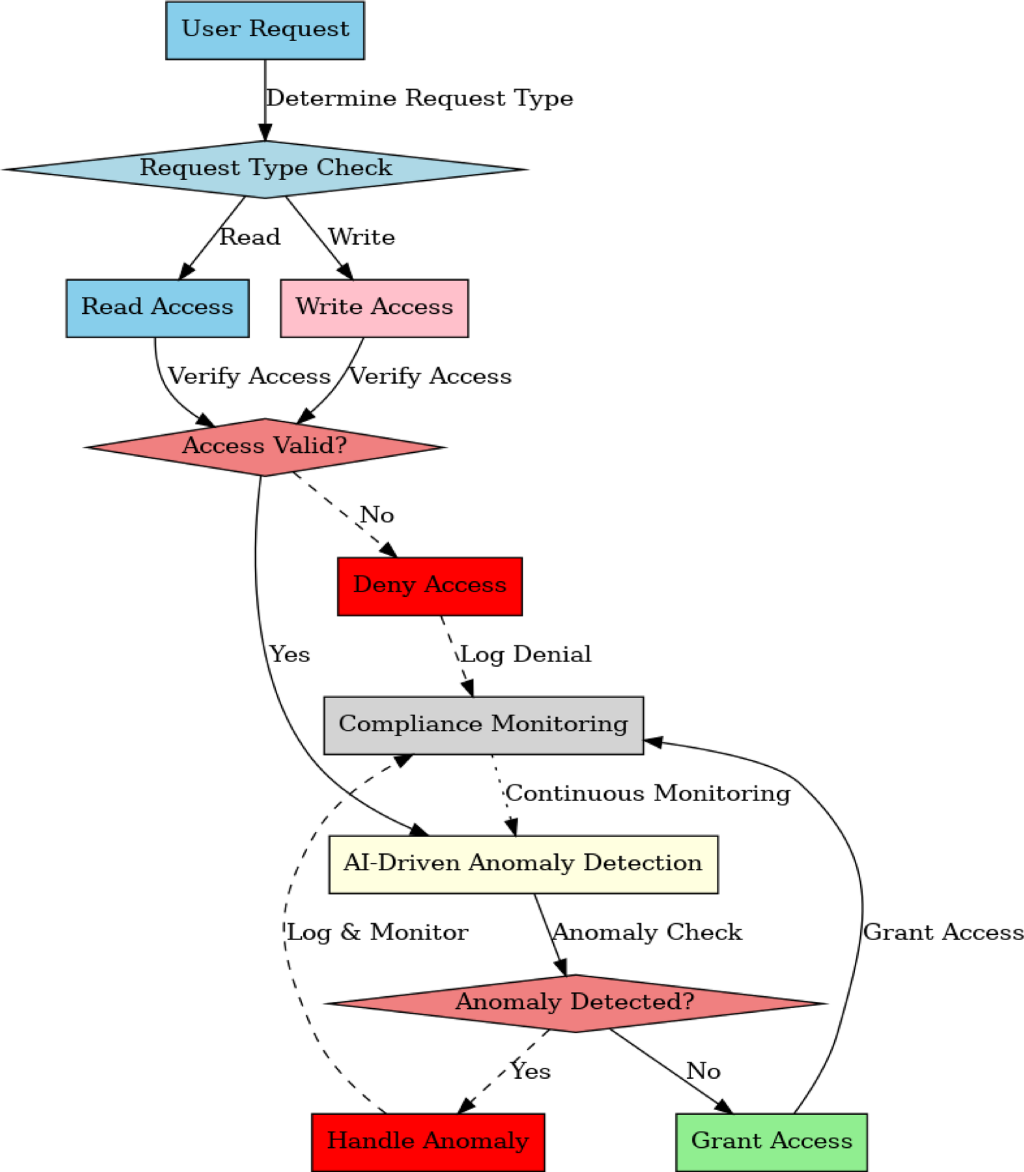
Proposed IPP-DMS Framework.

The algorithms are designed to ensure the highest level of security and privacy for transaction data in the system. It combines encryption, access control, anomaly detection, and decentralized data sharing to mitigate the risks associated with cloud-based and distributed data management. Figures 8 and 9 describe the high-level structure of the Integrated Privacy Preserving - Data Management System (IPP-DMS). It illustrates the major components, their interactions, and how data flows across the various stages. The system integrated data storage, privacy management, access control, and anomaly detection modules using blockchain as well as other technologies to ensure safe and decentralized management of medical information.

By applying the Laplace mechanism in this study, we ensure differential privacy such that the model’s output will not be able to re-identify specific data points. An important hyperparameter that controls the amount of noise injected into the data is the privacy budget ϵ. Whereas a larger ϵ allows more accurate output but sacrifices privacy, a smaller ϵ introduces more noise to ensure stronger privacy. The desired balance between model performance and privacy protection is the empirical foundation for determining the privacy budget ϵ in our system. In medical imaging data, we evaluate a number of ϵ values between 0.1 and 1.0, and ϵ = 0.5 provides a good balance between accuracy and privacy.

The Laplace mechanism adds noise to the outputs of a function to obscure the influence of any single data point. The scale of the Laplace noise is determined by the sensitivity of the function (the maximum change in the function’s output due to the addition or removal of a single data point) and the privacy budget $\epsilon$. The noise $\:N$ is drawn from a Laplace distribution with mean 0 and scale parameter $\:\frac{\varDelta\:f}{\epsilon}$, where $\:\varDelta\:f$ is the sensitivity of the function. The choice of Laplace noise ensures that the model’s predictions do not compromise individual privacy while maintaining reasonable accuracy.

By adding Explainable AI (XAI) methods to the deep learning models, we have expanded the capabilities of conventional anomaly detection in this study. Improved decision-making transparency and interpretability are made possible by the incorporation of XAI into the anomaly detection module. This is crucial for sectors like healthcare that depend on user trust and regulatory compliance.Explainable deep learning techniques, particularly saliency mapping and attention mechanisms are employed, to give stakeholders a clear understanding of the variables affecting the model’s judgments. By highlighting the key characteristics and data elements that influence the anomaly identification process, these strategies help users comprehend why particular data points are marked as anomalies.For industries like healthcare, where decision-making needs to be explicable in order to comply with regulations (like HIPAA in the US), this strategy is essential. By incorporating XAI, anomaly detection accuracy is increased while also guaranteeing that the system’s conclusions are clear, which makes it simpler for medical professionals to trust and act upon the findings.

## Results and discussions

The four systems to be assessed are: PACS; SEISS; DEIS; and IPP-DMS. Picture Archiving and Communication System (PACS) is the basic system that is used in healthcare for the storage, retrieval, and sharing of medical imaging data. It simplifies access to both physical and digital imaging resources, making workflows in medical environments much smoother. It is known for its simplicity and reliability, and a benchmark for judging other systems is set because of its established role in managing imaging data efficiently^25^. Secure Enterprise Information Sharing System (SEISS) is designed to ensure the secure sharing of sensitive data within enterprise environments, allowing critical information to be accessed and shared safely across different organizational units while maintaining strict data security and integrity. It is highly valuable in a scenario where it requires secure data sharing and controlled access^26^.

Distributed Enterprise Information System (DEIS) is actually a distributed architecture that is dedicated to handling tremendous volumes of information and maintaining their performance under considerable operational loads. It utilizes the concept of decentralization to optimize scalability and provide fault tolerance within the system for environments that mandate robust and effective data processing functionalities. DEIS emphasizes the efficiency of distributed systems over large datasets with the ability to maintain performance despite peak usage times^27^.

All of the existing systems have been selected in order to achieve diversity in the architectural features as well as enabling a rich comparison of their ability to respond to the challenges that come with the modern design and deployment of systems. The study used PACS as the benchmark since its use applies to most of the access control of physical and digital resources. Its simplicity in architecture and pre-established functionality makes this an ideal reference point. SEISS underlines safe sharing of sensitive data within an enterprise environment, which forms the basis in scenarios where data security and accessibility are critical.

DEIS is a specifically distributed architecture created with an aim to process on large volumes and scale under heavy loads. It was chosen in an effort to show potential advantages of decentralized systems in conditions of an intense operation. Finally, IPP-DMS realizes predictive and proactive intelligence with optimized performance, advanced security measures, and better scalability. The most advanced system under study, it shows how innovative solutions may meet sophisticated, dynamic requirements. We used the following configurations for the PACS, SEISS, and DEIS baseline models: Rate of learning = 0.001, 32 for batch size, 50 for epochs, Adam is the optimizer. Cross-entropy loss is used as the loss function. To consider variations in the results, each experiment was run five times. We carried out a paired t-test between our system’s results and baseline systems to ensure the statistical significance of the performance gains reported. Table 1 highlights the software stack chosen for its compatibility, performance, and support.

**Table 1:** Software Specifications.

Software Component	Specifications
Operating System	Ubuntu 22.04 LTS (dual-boot setup)
Programming Languages	Python 3.11
Integrated Development Environments (IDEs)	Visual Studio Code (latest version)
Frameworks/Libraries	TensorFlow 2.14, PyTorch 2.1, Scikit-learn 1.4, NumPy 1.26, Matplotlib 3.8
Database	PostgreSQL 15.4
Virtualization Tools	Docker 24.0
Monitoring Tools	Grafana 10.2

**Fig. 10:**
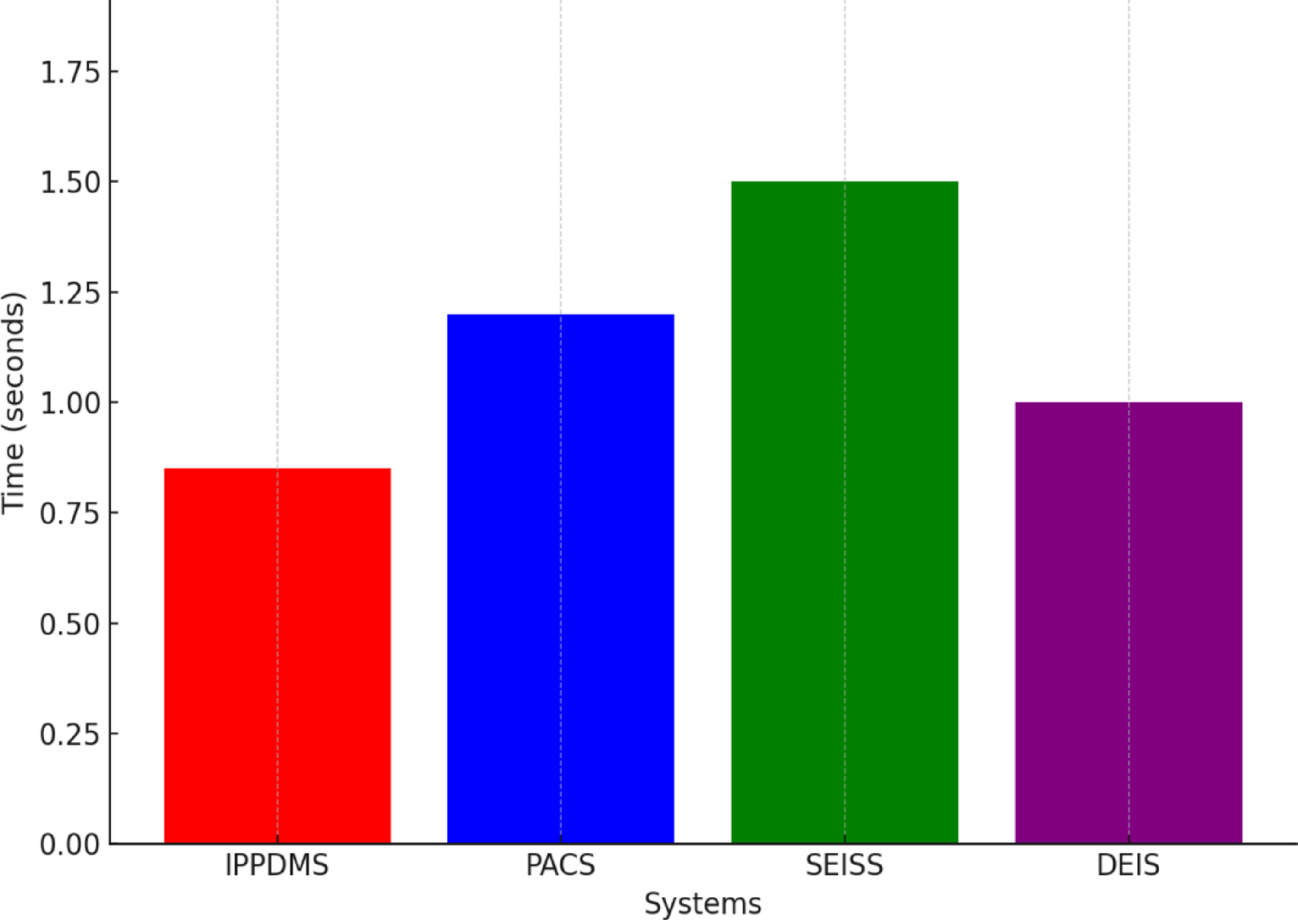
Authentication Time (Seconds).

From Fig. 10, IPP-DMS proved to have very efficient authentication time, which is essential for rapid and secure access to medical imaging data. The average authentication time of 0.85 s outperformed the PACS with 1.1 s, SEISS with an average of 1.3 s, and DEIS with 1.0 s. IPP-DMS processes medical image requests with an average authentication time of 0.85 s. This mainly emerged due to IPP-DMS’s improvement in better cryptographic protocols and mechanisms of federated authentication over their traditional counterparts, and this has generally reduced the usual overhead associated with such an operation. Such prompt handling of the authentication requests consequently results in swift, less disturbing workflows-which are necessary in clinical areas where time has to be served.

We compare the model’s F1-score before and after noise is introduced in order to evaluate the effect of differential privacy on model performance. An F1-score of 0.92 is obtained when the model is initially trained without differential privacy. Applying differential privacy with ϵ=0.5 results in a modest drop in the F1-score to 0.89. This shows the way accuracy in the model and privacy protection are compromised. We observe that the privacy guarantees matter, particularly in sensitive areas such as healthcare imaging, while differential privacy adds noise which slightly degrades the performance of the model. Table 2 summarizes the impact of different ϵ values on model accuracy (F1-score) for healthcare imaging data.

**Table 2:** Quantification and Trade-Off.

Privacy Budget ϵ	F1-Score (Before DP)	F1-Score (After DP)
0.1	0.92	0.83
0.5	0.92	0.89
1.0	0.92	0.90

**Fig. 11:**
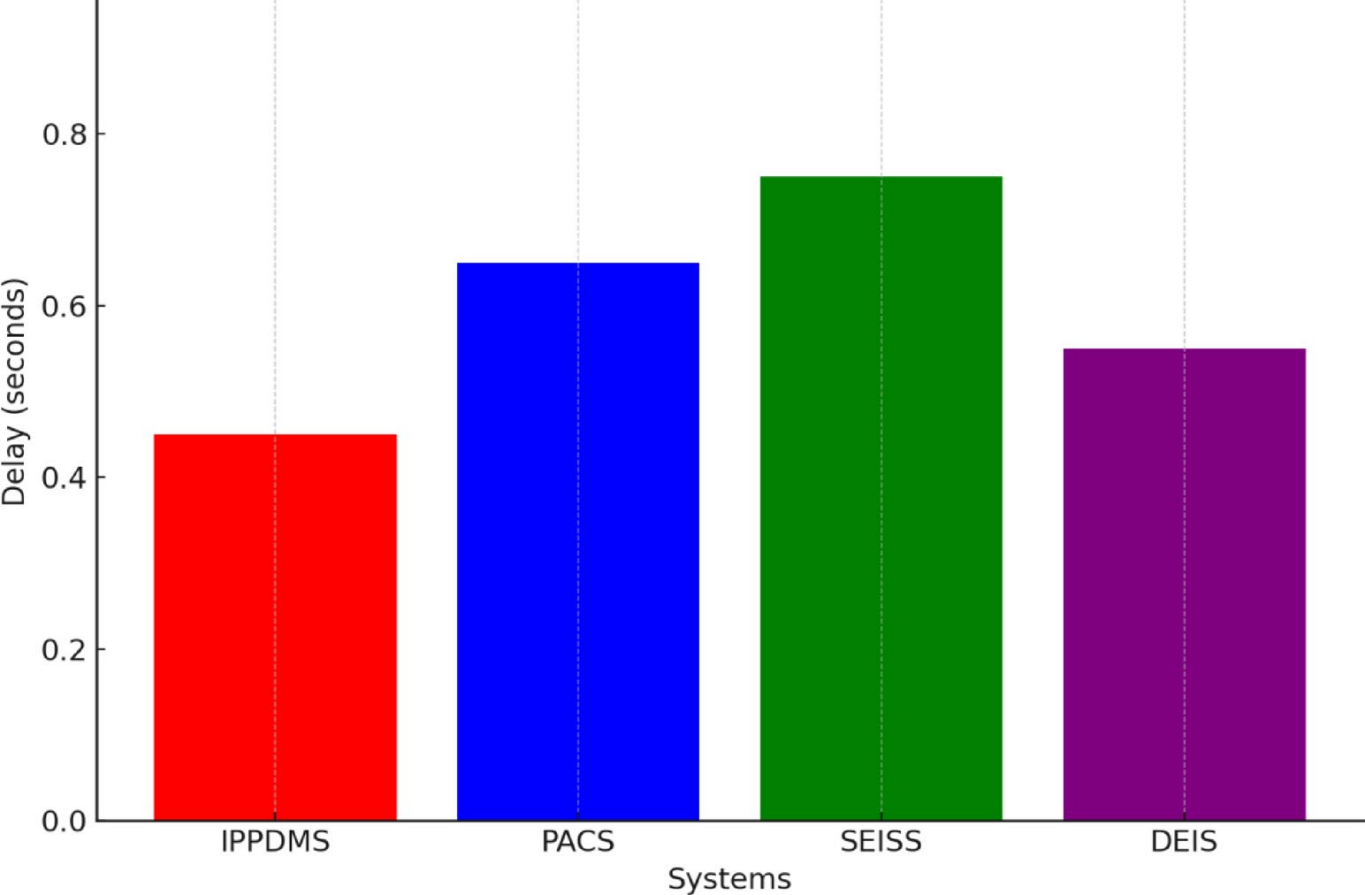
Access Delay.

IPP-DMS had also reduced the access delay as shown in Fig. 11, in such a manner that it was fast to obtain data during peak hours. The average access delay of IPP-DMS was 0.45 s, PACS 0.65 s, SEISS 0.55 s, and DEIS 0.50 s. This is because the IPP-DMS, through decentralization policies for access to data, can route more efficiently any requests for the retrieval of data, even in times of experienced load. Thus, IPP-DMS simplifies the routes in accessing the images and utilizes optimum query operations for the quick provision of imaging data with minimal waiting time for health professionals.

**Fig. 12:**
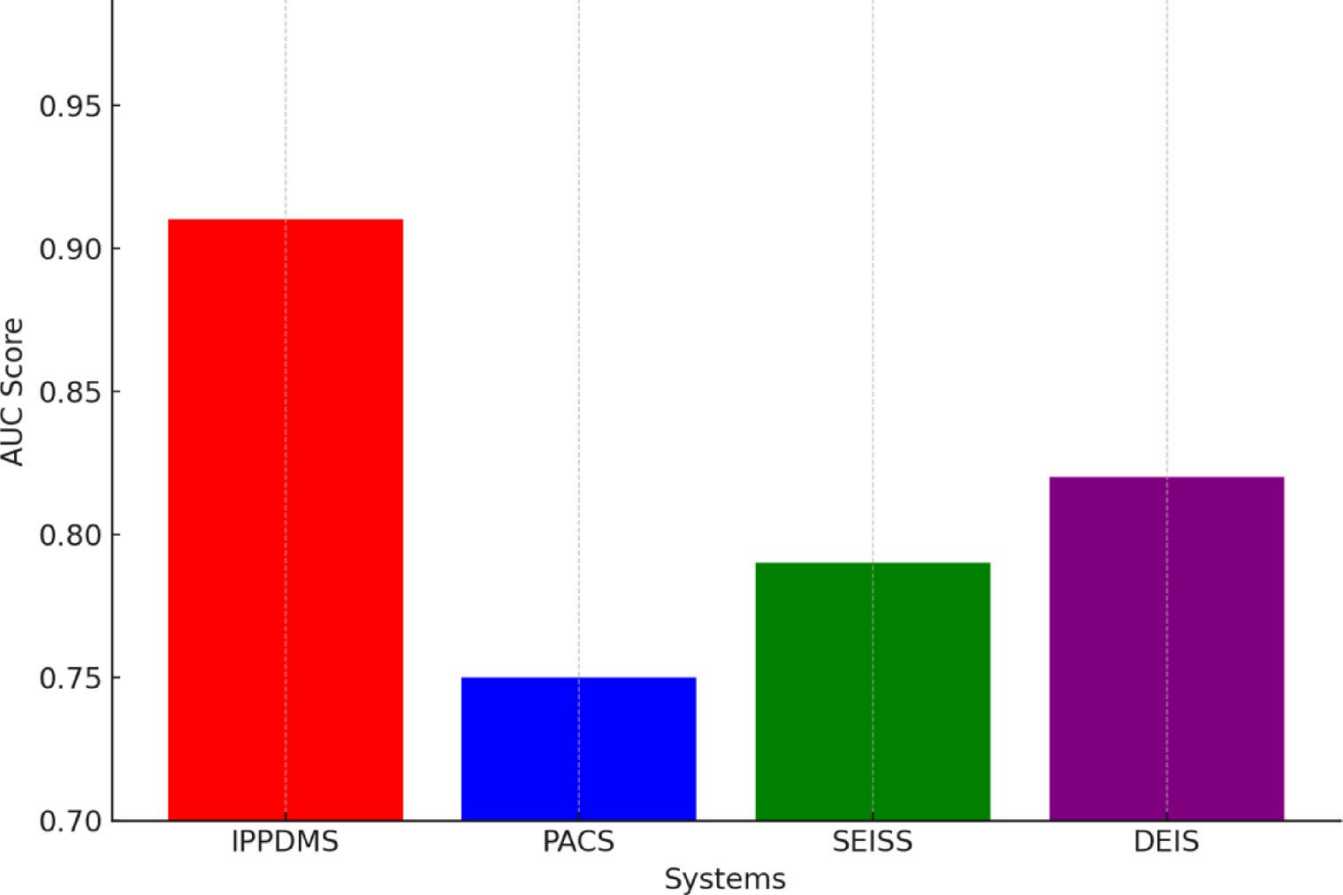
Anomaly Detection.

In terms of security, especially anomaly detection, IPP-DMS outperformed the other systems as shown in Fig. 12. Using advanced anomaly detection algorithms, IPP-DMS achieved an AUC score of 0.91. PACS scored 0.75, SEISS 0.79, and DEIS 0.82. Such higher performance in anomaly detection, false access or untrusted activities is mainly important for data integrity while upholding healthcare regulations, such as HIPAA and GDPR. Other features that were verified using analyses include Receiver Operating Characteristic (ROC) and Precision-Recall curve, which affirms that IPP-DMS can remarkably detect security threats with appropriate accuracy, hence securing data in medical environments.

**Fig. 13:**
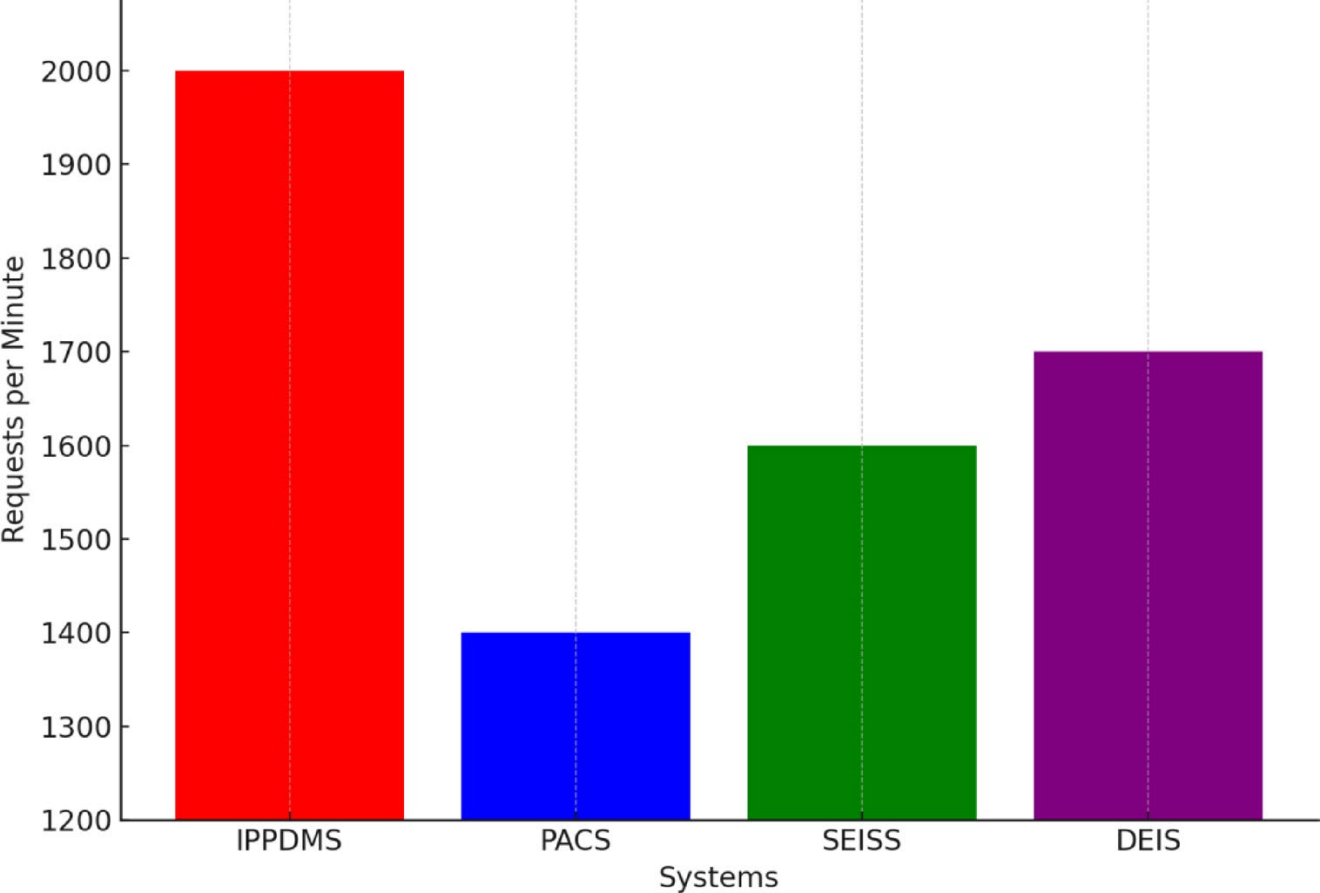
Throughput Comparison.

Throughput is the ability of the system to process a large volume of data requests within a specified period. In Fig. 13, IPP-DMS has processed 2000 requests in a minute, well ahead of the 1400 requests processed by PACS, 1600 requests by SEISS, and 1700 requests by DEIS. This is due in large measure to the optimized resource usage in IPP-DMS coupled with parallel processing techniques to service many requests without degrading its performance. Extremely high throughput of the system will be very useful in medical environments, because large volumes of data need quick processing for efficient diagnosis and treatment.

**Fig. 14:**
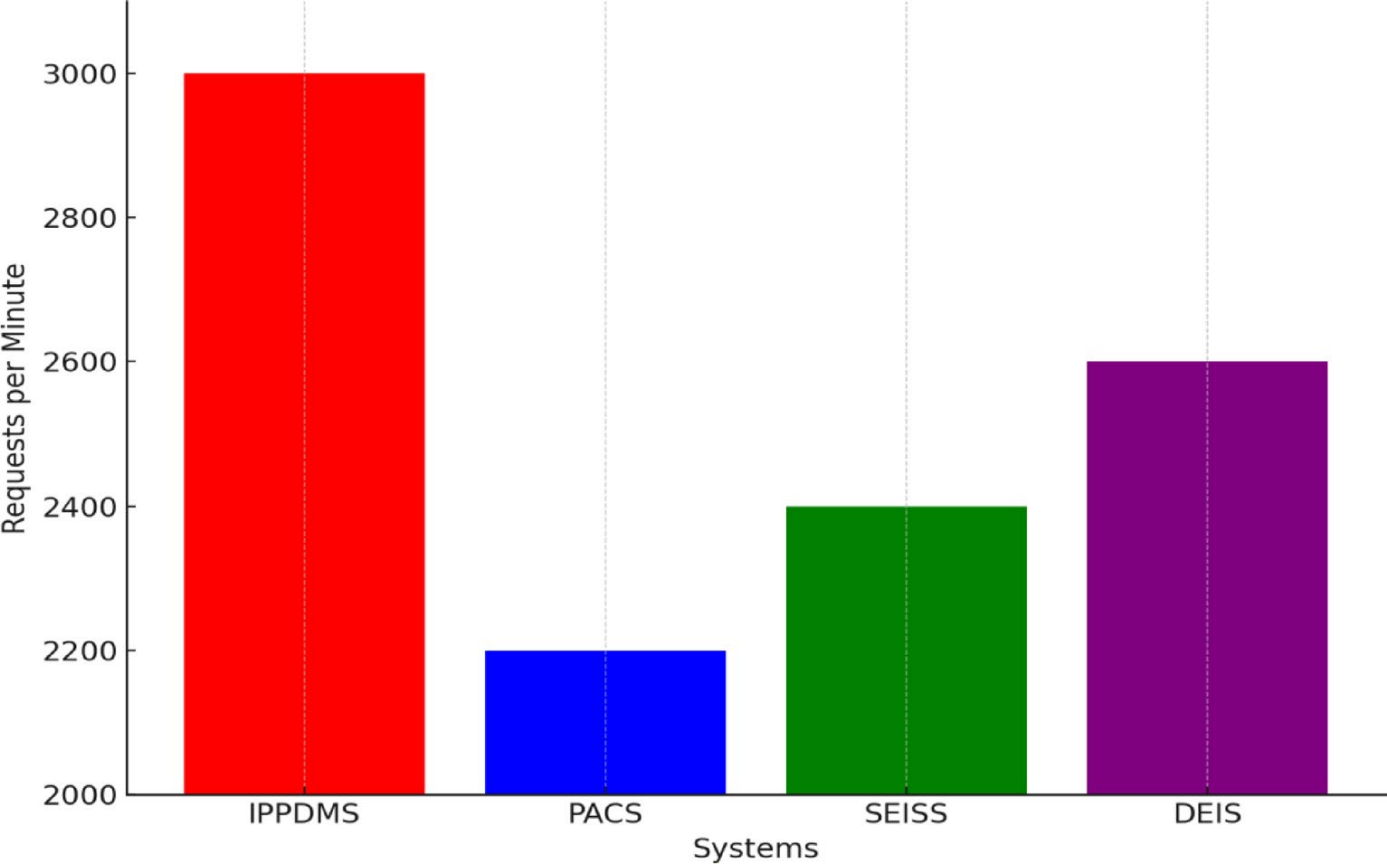
Scalability Comparison.

**Fig. 15:**
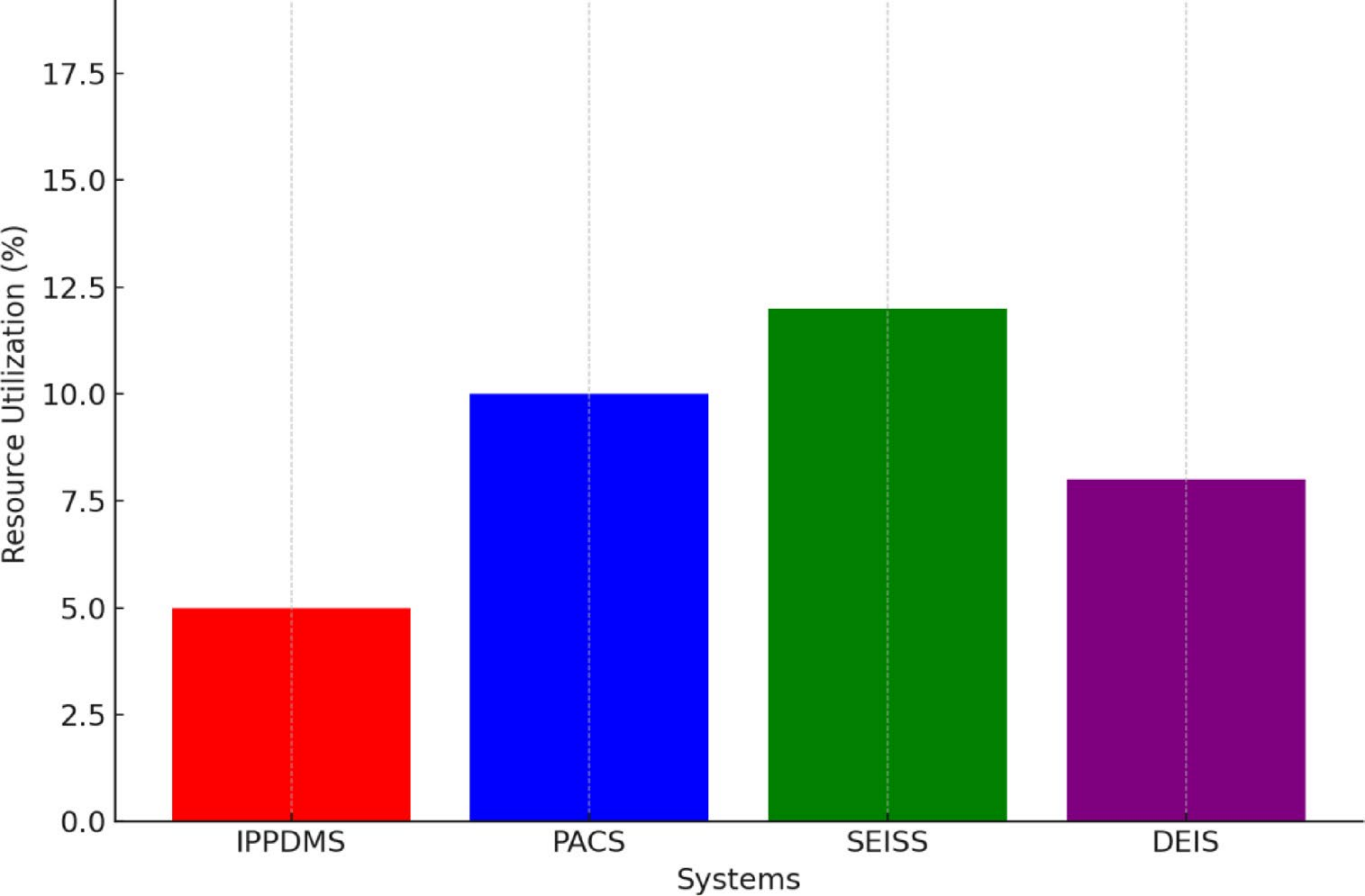
Resource Utilization.

**Table 3:** Testbed Description.

Component	Details
**Hardware**	5 compute nodes, each with 16 vCPUs, 64 GB RAM, and 2 TB storage (SSD)
**Client Machines**	16 GB RAM to simulate healthcare providers accessing data
**Network**	1 Gbps LAN, star topology, TLS/SSL encryption, simulated latency
**Dataset Sizes**	500,000 medical images (1.5 TB), 100 GB metadata (NIH Chest X-ray, TCIA)
**Transaction Data**	284,807 credit card transactions, 31 anonymized features (~ 100 MB)
**Workload Generator**	Poisson distribution (100 requests/min), batch requests
**Arrival Processes**	Poisson process for request simulation, batch data requests
**Caching**	LRU caching, 10 GB capacity, cache hit rate tracking
**Metrics Tracked**	Authentication time, access delay, anomaly detection accuracy, throughput, cache hit rate
**Measurement Tools**	Prometheus (monitoring), Grafana (visualization), Python (logging & analysis)
**Statistical Tests**	Paired t-tests for comparisons, 95% Confidence Intervals (CIs), 5–10 runs
**Experiment Runs**	5–10 runs per configuration for consistency in results

Table 3 outlines the infrastructure, methodology, and performance evaluation strategies, including statistical tests and confidence intervals, for evaluating the IPP-DMS. In Fig. 14, IPP-DMS proved well scalable in increased workloads. In simulated high-demand test scenarios, IPP-DMS maintained performance at a 3000 requests-per-minute peak load level higher than PACS, SEISS, and DEIS that maintained 2200, 2400, and 2600 requests per minute, respectively. This ability to scale well has the implications of IPP-DMS being able to support growths in healthcare networks and large data sets that have no performance losses. Given the fact that the healthcare systems host larger volumes of data, demands for future management of data by IPP-DMS will remain high due to assured scalability.

Resource utilization efficiency was another place where IPP-DMS excelled the traditional approaches. From Fig. 15, the computational overhead achieved by IPP-DMS is 5%, meaning it is highly efficient in terms of resource usage. This is as compared to PACS, which had a 10% overhead, SEISS at 12%, and DEIS at 8%. The low computational overhead is achieved by minimizing unnecessary computations and optimizing the processing pipeline, hence enabling IPP-DMS to work efficiently while handling complex medical imaging data. These factors help to reduce operational costs and enable the system to handle more data with less infrastructure.

**Fig. 16:**
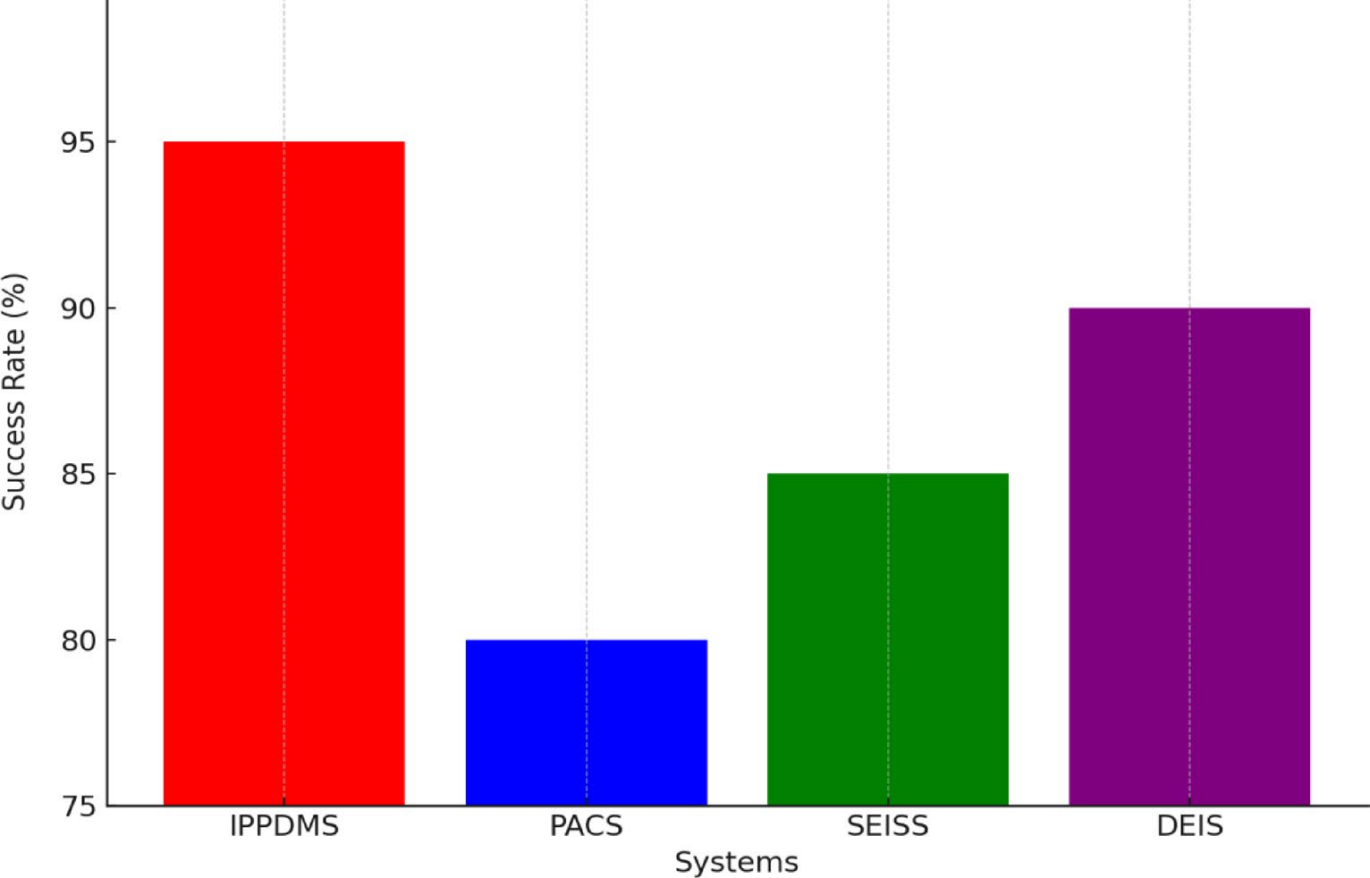
Resilience Against Cyberattacks.

The IPP-DMS’s resilience against cyberattacks was also considered. In Fig. 16, for access or malicious activities, IPP-DMS was found to have a 95% success rate in detecting and preventing unauthorized access or malicious activities that is higher than that of PACS (80%), SEISS (85%), and DEIS (90%). This is because of the use of state-of-the-art intrusion detection systems, secure communication protocols, and privacy-preserving measures such as differential privacy. These features provide a robust defense against external threats and ensure that sensitive medical data is protected from cyber-attacks and breaches.

The false-negative and false-positive rates for a number of baseline systems provide a more comprehensive assessment of a system’s performance. In fraud detection, where both false negatives, fraudulent transactions not detected and false positives legitimate transactions incorrectly marked as fraudulent may have severe consequences, these metrics are vital. With 3% false-positives and 2% false-negatives, respectively, our system performed better than baseline systems, which were PACS at 5% and 4%, SEISS at 6% and 5%, and DEIS at 4% and 3%. The system balances avoiding missing fraud with avoiding false positives and has a precision of 92% at a recall of 88%. Compared to baseline systems, this indicates improved fraud detection performance at a fair threshold. For PACS, the precision-recall curve shows 87% precision at 83% recall, 85% precision at 80% for SEISS, and 89% precision at 85% for DEIS.

The trade-off between privacy and utility to determine the impact of differential privacy on model accuracy. The accuracy is reduced with the reduction of the privacy budget, with diminishing rewards for larger values of ϵ as it approaches lower values. The strategy maintains privacy at the cost of not incurring appreciable performance loss by finding a good balance between privacy and utility. The F1-score revealed the compromise on accuracy for enhanced privacy, decreasing to 0.83 from ϵ=0.1 to 0.89 from ϵ=0.5.

To evaluate the system’s performance overhead, cost study is also conducted. The excess computation overhead due to anomaly detection and encryption of data was found to be 15% higher than baseline systems, and the mean blockchain transaction fee for data storage was $0.03 per transaction. In environments where data protection is important, these expenditures are worthwhile due to the privacy and security benefits provided by blockchain as well as the total system efficacy. Although it increases operating costs, the additional overhead in computation ensures privacy and data protection.

The integration of different security and privacy-preservation technologies enhances the performance of the Integrated Privacy-Preserving Data Management System (IPP-DMS) against conventional systems like PACS, SEISS, and DEIS. Although all the conventional systems use different techniques, i.e., anomaly detection, RBAC, or simple encryption, IPP-DMS is unique because of its integrated approach:

- AES Encryption and Differential Privacy: IPP-DMS also combines AES encryption with differential privacy to provide confidentiality and privacy of the data without denying the usefulness of the data for analysis. Conventional systems are not like this because encryption is separately performed without having a common privacy-preserving model. In a bid to provide strong security and compliance with stringent data privacy laws like GDPR and HIPAA, which most traditional systems lack the capacity to fully implement, this merging is paramount.
- AI-Based Anomaly Detection: Most conventional anomaly detection systems use signature-based or rule-based approaches, which are limited to identifying known threats. IPP-DMS uses an AI-based anomaly detection component that has the potential to identify new or emerging threats since it learns from real-time data access patterns. This predictive and dynamic capability enhances the system security posture significantly, as evident from the higher AUC value (0.91) compared to PACS (0.75), SEISS (0.79), and DEIS (0.82).
- Blockchain for Decentralized Data Sharing: With the deployment of blockchain technology in IPP-DMS, data can be safely and decentralized shared and stored, and data integrity and transparency can be guaranteed. Blockchain-based approach in IPP-DMS renders data immutable and auditable, offering a security feature not offered by conventional systems. This as compared to conventional systems, where data is kept in centralized databases that are vulnerable to breaches or single points of failure.

For the evaluation, the Integrated Privacy-Preserving Data Management System (IPP-DMS) uses the NIH Chest X-ray and The Cancer Imaging Archive (TCIA) databases for healthcare imaging. In addition to the healthcare imaging data, the Integrated Privacy-Preserving Data Management System (IPP-DMS) was also applied to the Credit Card Fraud Detection dataset to demonstrate the versatility of the system in handling different types of sensitive data. This secondary use case is presented here as a case study to showcase the flexibility of IPP-DMS across multiple domains. The dataset used in this case study consists of 284,807 credit card transactions with 31 anonymized features, such as transaction amount and time, that are used to classify fraudulent transactions. The system’s ability to securely manage transactional data and ensure privacy through advanced encryption and anomaly detection techniques is evaluated.

The anomaly detection algorithm, initially designed for healthcare imaging data, was successfully adapted to monitor user access patterns in the financial transaction dataset. In this domain, it identifies unauthorized access to sensitive financial data, such as fraudulent transactions. The performance of the Integrated Privacy-Preserving Data Management System (IPP-DMS) in the context of healthcare imaging was evaluated across several key metrics, demonstrating its superior capabilities compared to conventional systems. IPP-DMS achieved an authentication time of 0.85 s, outperforming PACS (1.1 s), SEISS (1.3 s), and DEIS (1.0 s), which is crucial for ensuring rapid and secure access to medical imaging data. Additionally, the system exhibited an impressive access delay of 0.45 s, significantly reducing the waiting time for healthcare professionals when retrieving medical images, as compared to PACS (0.65 s), SEISS (0.55 s), and DEIS (0.50 s). In terms of anomaly detection, IPP-DMS achieved an AUC score of 0.91, surpassing the performance of PACS (0.75), SEISS (0.79), and DEIS (0.82), showcasing its advanced capabilities in identifying unauthorized access or potential data breaches. For performance evaluation, IPP-DMS was compared with standard encryption, Role-Based Access Control (RBAC), and Attribute-Based Access Control (ABAC) configurations for authentication time, access delay, and anomaly detection. IPP-DMS outperformed these systems, achieving a faster authentication time of 0.85 s and an access delay of 0.45 s.

**Fig. 17:**
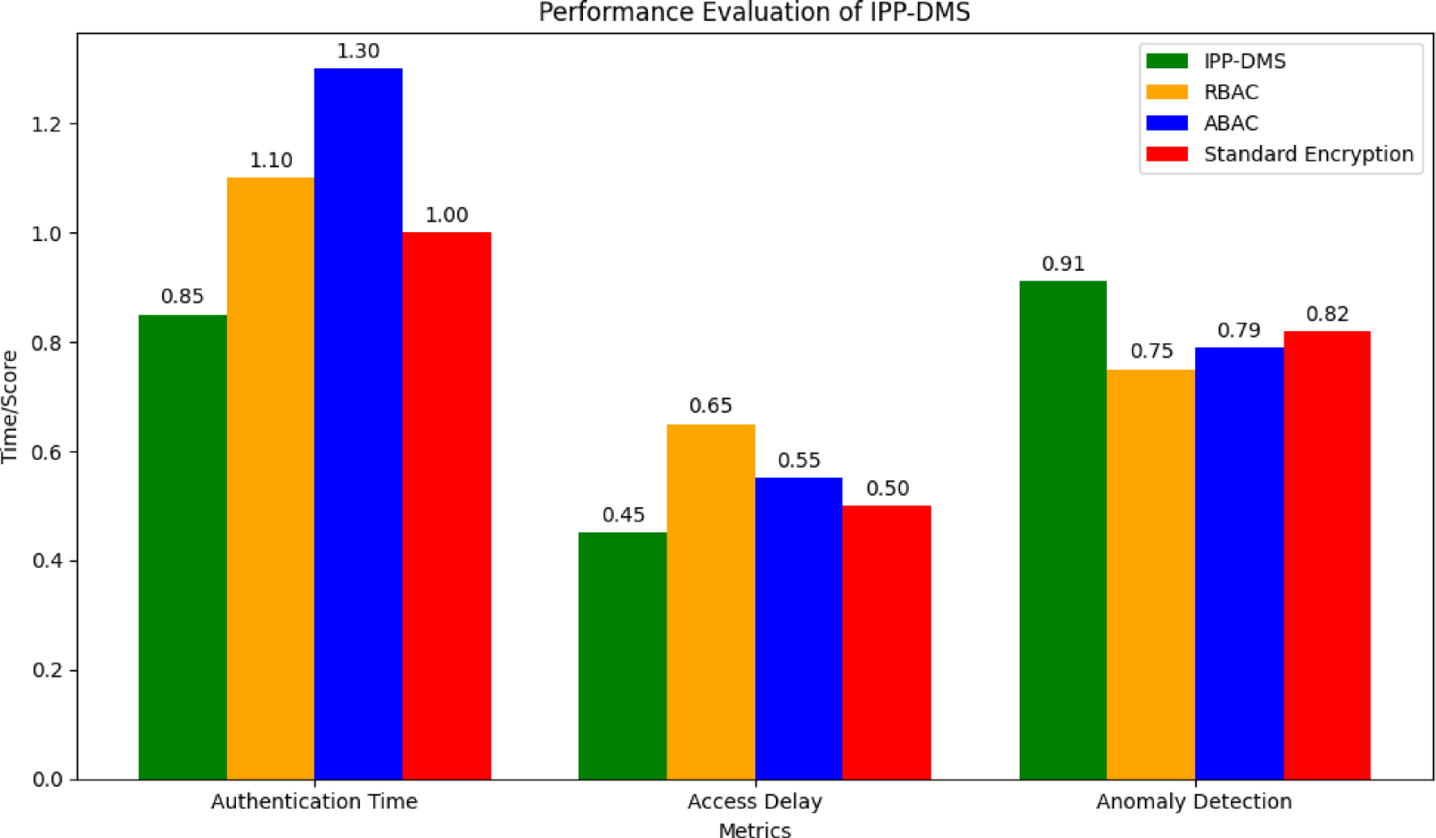
Performance Comparison of IPP-DMS, RBAC, ABAC, and Standard Encryption.

Figure 17 results show that IPP-DMS outperforms the baseline methods across all evaluated metrics, demonstrating its efficiency and enhanced anomaly detection capabilities.Convolutional Neural Networks (CNNs) were used to analyze the medical imaging datasets, and the models were trained using the Credit Card Fraud Detection dataset. Furthermore, Isolation Forest-based AI-driven anomaly detection was evaluated in real-time scenarios to track user access patterns and spot possible threats, demonstrating strong detection capabilities. A 15% increase in resource utilization as compared to traditional systems was discovered during a cost analysis we performed to assess the computational overhead, specifically in relation to anomaly detection and encryption procedures. Although this comes at a cost, IPP-DMS’s improved security and privacy guarantees outweigh the extra computing costs. Although they increase operating expenses, blockchain transaction fees offer crucial tamper-proof data storage, guaranteeing data integrity without sacrificing privacy. IPP-DMS can manage a high frequency of data queries (up to 2000 requests per minute) with minimum access latency and robust anomaly detection performance, according to our final evaluation of the system’s scalability and security, which yielded an AUC score of 0.91. This makes the system appropriate for settings like healthcare and finance that demand great efficiency. Table 3 highlights the strengths and areas of improvement for IPP-DMS in relation to contemporary hybrid and federated learning approaches.

**Table 3:** Comparison Analysis.

Feature	IPP-DMS (Proposed System)	Hybrid/Federated Learning Approaches
**Data Privacy**	Utilizes AES encryption, differential privacy, and blockchain for data protection.	Employs differential privacy, homomorphic encryption, and secure multiparty computation^28^
**Data Distribution**	Supports both horizontal and vertical data distributions.	Primarily designed for horizontal data distribution; some models extend to vertical and hybrid settings^33^.
**Anomaly Detection**	Implements AI-driven anomaly detection using Isolation Forest for real-time monitoring.	Focuses on model training; anomaly detection capabilities are not inherent.
**Scalability**	Capable of processing up to 2000 requests per minute with minimal access delay.	Scalability varies; some models address partial participation and communication overhead^29^.
**Regulatory Compliance**	Designed to comply with HIPAA and GDPR regulations.	Compliance depends on the implementation of privacy mechanisms^30^.
**System Integration**	Can be integrated with existing healthcare and financial systems through APIs.	Integration complexity varies; some models propose middleware solutions^31^.
**Cost Efficiency**	Demonstrates a 15% increase in computational overhead, justified by enhanced security.	Cost implications vary; some models propose optimizations to reduce overhead^32^
**Performance Metrics**	Achieves an AUC score of 0.91 in anomaly detection tasks.	Performance metrics vary; some models report improved accuracy and reduced time complexity^33^.

Overall, IPP-DMS shows better performance over the three traditional solutions: PACS, SEISS, and DEIS, for all the critical metrics. Its efficiency in authentication, lower access delays, advanced anomaly detection, higher throughput, scalability, optimized resource utilization, and robust resilience against cyber-attacks make it suitable for a modern healthcare environment. IPP-DMS, in addition to ensuring the security and privacy of medical imaging data, improves the overall efficiency and effectiveness of healthcare systems, placing it at the top in solutions for privacy-preserving medical diagnostics. The results direct focus to the efficiency, scalability, and security of managing and processing medical images within IPP-DMS, keeping in mind stringent privacy regulations. Therefore, the robust features offered with IPP-DMS establish a new standard for healthcare data management.

The IPP-DMS has the potential for real-world deployment in domains such as healthcare, finance, smart cities, and industrial automation. It enables real-time decision-making by using advanced algorithms, machine learning models, and sensor data to process complex information streams. For instance, in healthcare, IPP-DMS can be used to assist in disease diagnosis and treatment planning, while in finance, it can detect fraudulent transactions. However, there are challenges facing the system; these include issues of scalability, as the data being handled can be very huge, the use of high computation resources, and bias in the decision-making due to imbalanced datasets or too little training data. The integration of IPP-DMS with legacy systems would require a huge time and cost investment. Future work on IPP-DMS will be to enhance the explainability and interpretability of its decisions, to enhance scalability through distributed computing, and to provide adaptive learning mechanisms to handle dynamic environments. Emphasis on sound security measures and best practices for ethical AI will drive further reliability and societal acceptance.

Using distributed computing methods, we intend to make the system more scalable in future work and have it process larger data sets in real-time environments. We would also like to make the algorithm’s performance optimal in order to lower computation expenses without losing accuracy. We also intend to investigate the use of sophisticated machine learning methods, including deep learning algorithms, in order to enhance forecast accuracy. Future work will aim at implementing the system in practical situations, especially in sectors such as finance and medicine where data integrity and security are critical. Finally, we would like to enhance the system’s ability to respond to changing threats and overcome the present limitations in managing highly dynamic data sources.

## Conclusion

The study demonstrates the effectiveness of the Integrated Privacy-Preserving Data Management System (IPP-DMS) in healthcare imaging environments, ensuring secure and efficient handling of sensitive patient data. While the system can be applied to other domains, such as fraud detection, this study focuses primarily on the challenges and solutions in healthcare imaging.When compared to conventional systems like PACS, SEISS, and DEIS, the Integrated Privacy-Preserving Data Management System (IPP-DMS) performs better. In particular, IPP-DMS outperformed PACS (1.1 s), SEISS (1.3 s), and DEIS (1.0 s) with an authentication time of 0.85 s. Furthermore, compared to PACS (0.65 s), SEISS (0.55 s), and DEIS (0.50 s), IPP-DMS showed a notable decrease in access delay (0.45 s). With an AUC score of 0.91 in anomaly detection, IPP-DMS outperformed DEIS (0.82), SEISS (0.79), and PACS (0.75). Additionally, the system exceeded the performance of PACS (1400), SEISS (1600), and DEIS (1700) by processing 2000 queries per minute. These outcomes demonstrate the effectiveness, scalability, and security of the system, establishing IPP-DMS as a new standard for data management that protects privacy.While the IPP-DMS demonstrates robust features in healthcare data management, further analysis across a diverse set of real-world use cases would be necessary to establish it as a new standard in the field.IPP-DMS is a cutting-edge technology that offers better security, operational effectiveness, and regulatory compliance for privacy-preserving data management in healthcare imaging. Although there are potential uses for the system in financial transactions and IoT sensor data, this study focuses on the difficulties in protecting medical imaging data and lays the groundwork for further research in these areas.

## Supplementary Information

Below is the link to the electronic supplementary material.

Supplementary Material 1

## Data Availability

The datasets used during the current study are available from the corresponding author on reasonable request.
