# Supplementary material for: IPP-DMS: A scalable privacy-preserving data management system for secure and efficient handling of large-scale datasets
Source: Sci Rep. 2025 Dec 27;16:2583. doi: 10.1038/s41598-025-32498-6 (PMC12820145; doi:10.1038/s41598-025-32498-6)
Supplement: Supplementary file 1 [file 41598_2025_32498_MOESM1_ESM.odt]

### Appendix A: Smart Contract Code, Deployment Details, and Threat Model

Smart Contract Code

The code allows for the secure storage and retrieval of healthcare metadata while maintaining privacy through data anonymization techniques like hashing.

// SPDX-License-Identifier: MIT

pragma solidity ^0.8.0;

contract PrivacyPreservingDataManagement {

    // Struct to represent healthcare metadata

    struct HealthcareData {

        uint256 dataId;

        string dataHash;  // Hash of the healthcare data for privacy

        address owner;    // Owner of the data

        uint256 timestamp; // Timestamp of the data entry

    }

    // Mapping to store healthcare data using dataId

    mapping(uint256 => HealthcareData) public healthcareData;

    // Event to log data storage

    event DataStored(uint256 dataId, address indexed owner, uint256 timestamp);

    // Modifier to ensure only the owner can update the data

    modifier onlyOwner(uint256 dataId) {

        require(healthcareData[dataId].owner == msg.sender, "You are not the owner");

        _;

    }

    // Function to add healthcare data

    function addHealthcareData(uint256 dataId, string memory dataHash) public {

        require(healthcareData[dataId].dataId == 0, "Data ID already exists");

        healthcareData[dataId] = HealthcareData({

            dataId: dataId,

            dataHash: dataHash,

            owner: msg.sender,

            timestamp: block.timestamp

        });

        emit DataStored(dataId, msg.sender, block.timestamp);

    }

    // Function to retrieve healthcare data by ID

    function getHealthcareData(uint256 dataId) public view returns (string memory dataHash, address owner, uint256 timestamp) {

        require(healthcareData[dataId].dataId != 0, "Data not found");

        HealthcareData memory data = healthcareData[dataId];

        return (data.dataHash, data.owner, data.timestamp);

    }

    // Function to update healthcare data (only by the owner)

    function updateHealthcareData(uint256 dataId, string memory newDataHash) public onlyOwner(dataId) {

        healthcareData[dataId].dataHash = newDataHash;

    }

}

Deployment Details

IPP-DMS is deployed on a permissioned blockchain to ensure access control and data privacy. The network consists of multiple nodes, each running a copy of the smart contract. A permissioned network provides the benefit of maintaining confidentiality for healthcare data by restricting access to authorized users only. Gas costs are optimized for efficiency by limiting computation-heavy operations and using off-chain computations where necessary. The contract utilizes minimal gas for read operations (data retrieval) and higher gas consumption for write operations (adding or updating data).To handle potential congestion, we have implemented load balancing techniques to ensure that requests are distributed across nodes evenly. Additionally, transaction batching is used to group similar transactions together, reducing the network load and improving efficiency.

Threat Model

Attackers might attempt to gain unauthorized access to sensitive healthcare data. This is mitigated by using a permissioned blockchain, where access to the system is limited to authorized participants (healthcare providers, researchers). Exposure of sensitive data due to vulnerabilities in the blockchain or smart contract. This is mitigated by data anonymization, where sensitive data is stored off-chain and only hashes are stored on-chain. Potential flaws or bugs in the smart contract could be exploited. To mitigate this risk, the contract undergoes regular audits and security reviews before deployment.Attacks such as Sybil or 51% attacks can compromise decentralized networks. These threats are less likely on a permissioned blockchain, as control over the network is centralized.

Permissioned Chain vs. Public Layer 1 Blockchain

While public Layer 1 (L1) blockchains (such as Ethereum) are widely used, they are not well-suited for managing healthcare metadata.Public L1 blockchains expose all transactions, including sensitive healthcare data, to the public. Even though data can be hashed, the transparency of public chains is not ideal for privacy-sensitive applications like healthcare. Healthcare data must comply with privacy regulations such as HIPAA (in the U.S.) and GDPR (in the EU). A public blockchain does not provide the fine-grained access control needed to ensure compliance with these regulations.On the other hand, a permissioned blockchain offers several advantages:

- Only authorized participants (e.g., hospitals, healthcare providers, researchers) can access and modify the data, ensuring privacy and security.

- Permissioned blockchains can handle a higher throughput of transactions without the high gas fees typically associated with public L1 blockchains.

- The system allows secure and transparent audits without exposing sensitive data, which can be difficult to achieve on public chains without compromising privacy.

Measurable Benefits of a Permissioned Chain Over a Signed Audit Log

Unlike a signed audit log, where data might still be accessible if someone gains access to the log, a permissioned blockchain keeps sensitive metadata accessible only to authorized participants.

A permissioned blockchain can handle more transactions with lower costs compared to public blockchains, which suffer from higher gas fees and congestion.

The permissioned chain ensures that only authorized entities can access, modify, or store data, making it more secure for handling sensitive healthcare information.

Differential Privacy Mechanisms and Budget Allocation

In this study, we implement Differential Privacy (DP) using the Laplace Mechanism for numeric outputs and the Exponential Mechanism for categorical outputs. The Laplace Mechanism is used for perturbing numeric outputs, where noise is added proportional to the sensitivity of the function divided by the privacy budget.

Noise ~ Laplace(0, Δf / ε)

where Δf is the sensitivity of the function, and ε is the privacy budget. For categorical outputs, the Exponential Mechanism is applied, with the probability of selecting an output based on a utility function and privacy budget:

P[Output = a] = exp(ε * u(a) / 2Δu) / Z

where u(a) is the utility score of output a, Δu is the sensitivity of the utility function, and Z is the normalization factor.

The protected outputs in this study include predictions from fraud detection models, aggregated statistics (like means and sums), and sensitive features such as transaction amounts and timestamps. To compute the sensitivity of these outputs, we use the L2 norm of the weights for models like logistic regression or the range of values for aggregation operations. The sensitivity of the prediction in logistic regression is computed as:

Δf = || w ||₂

where w represents the model weights.

To account for the privacy budget across multiple tasks such as data preprocessing, model training, and prediction, we apply Advanced Composition for differential privacy. When multiple mechanisms are applied independently, the total privacy budget is the sum of the individual budgets, but using Advanced Composition ensures that repeated use of the privacy budget is correctly handled. 

εtotal = ln(1 + n * eε - 1)

where n is the number of tasks, and ε is the privacy budget allocated per task. In this study, the privacy budget is allocated across three tasks: preprocessing (ε₁ = 0.1), model training (ε₂ = 0.2), and prediction (ε₃ = 0.3), resulting in a total privacy budget of:

εtotal = 0.1 + 0.2 + 0.3 = 0.6

Code shows how to apply differential privacy to logistic regression predictions

from diffprivlib.models import LogisticRegression

from diffprivlib.mechanisms import Laplace

# Example of adding Laplace noise to logistic regression

epsilon = 0.1  # Privacy budget

sensitivity = 1  # Sensitivity of the logistic regression model

# Example data (X: features, y: target)

X = [[1, 2], [2, 3], [3, 4]]

y = [0, 1, 0]

# Create and train a differentially private logistic regression model

model = LogisticRegression(epsilon=epsilon)

model.fit(X, y)

# Predict with added Laplace noise

laplace = Laplace(epsilon=epsilon, sensitivity=sensitivity)

prediction = laplace.add_noise(model.predict(X))

print(prediction)

For formal and rigorous DP accounting, we recommend using established libraries such as diffprivlib (GitHub Link:[ ](https://github.com/IBM/diffprivlib)[https://github.com/IBM/diffprivlib](https://github.com/IBM/diffprivlib)), PySyft (GitHub Link:[ ](https://github.com/OpenMined/PySyft)[https://github.com/OpenMined/PySyft](https://github.com/OpenMined/PySyft)), and the Google Differential Privacy Library (GitHub Link:[ ](https://github.com/google/differential-privacy)[https://github.com/google/differential-privacy](https://github.com/google/differential-privacy)).

APPENDIX B : GDPR/HIPAA compliance plan table

| Compliance Area | Details | Remarks |
| --- | --- | --- |
| Roles for Data Access | Access is restricted to authorized personnel only, with defined roles and permissions for handling sensitive data. | Data Analyst: Read-only access to transaction data. Data Administrator: Full access to data processing, anonymization, and model training. |
| Data Protection Impact Assessment (DPIA) | A DPIA has been conducted to evaluate privacy risks and determine necessary mitigation strategies. | Conducted: January 2025- Risk Level: Low to Medium- Mitigation Measures: Enhanced access controls, encryption during data transfer. |
| Data Subject Rights | Rights include access, rectification, erasure, and restriction of processing of personal data. | Access Requests: Respond within 30 days. Deletion Requests: Data will be deleted within 60 days after request approval. |
| Logging and Monitoring | Comprehensive logs track data access and modifications, ensuring accountability and compliance. | Log Retention Period: 6 months. Log Review Frequency: Monthly. Access Logs Retained: Yes (for all data access events). |
| Data Retention | Data is retained only as long as necessary, with secure deletion or anonymization once retention periods have expired. | Retention Period: 5 years for transactional data. Deletion Method: Secure wipe and data anonymization post retention period. |
| Breach Handling | A protocol for promptly identifying, reporting, and mitigating data breaches, including notifications to authorities and affected individuals. | Breach Notification: Within 72 hours of discovery. Affected Individuals Notified: Yes (if personal data is exposed). Breach Response Plan: Immediate isolation, investigation, and remediation steps within 24 hours. |
